# Fast identification of optimal pure platinum nanoparticle shapes and sizes for efficient oxygen electroreduction†

**DOI:** 10.1039/c9na00252a

**Published:** 2019-06-03

**Authors:** Marlon Rück, Aliaksandr Bandarenka, Federico Calle-Vallejo, Alessio Gagliardi

**Affiliations:** Department of Electrical and Computer Engineering, Technical University of Munich 80333 München Germany alessio.gagliardi@tum.de; Physics Department, Technical University of Munich 85748 Garching Germany; Department of Materials Science and Physical Chemistry, Institute of Theoretical and Computational Chemistry (IQTC), University of Barcelona 08028 Barcelona Spain

## Abstract

Recent advances in experimental synthesis of nanostructures have shown that the interplay between nanoparticle shapes and sizes is crucial to achieve catalysts with high mass activity toward oxygen electroreduction. This is particularly important for proton-exchange membrane fuel cells (PEMFCs), in which expensive and scarce Pt electrocatalysts are used. In this work, we propose a theoretical approach for oxygen electroreduction on PEMFCs to identify not only the size of optimal nanoparticles, but also their shapes. Remarkably, high mass activities up to 4.28 A mg_Pt_^−1^ are predicted for rod-like nanostructures. Furthermore, we examine nanostructure size effects to guide chemical routes for experimental synthesis of the identified electrocatalysts. Our fast theoretical evaluation of thousands of different nanostructures aids in the search for active catalysts, as substantially enhanced mass activities over commercial Pt/C are predicted for pure Pt electrocatalysts, thus unveiling great potential to reduce the Pt loading in PEMFCs.

## Abstract 2

Unraveling size effects on the mass activity guides chemical routes for synthesis of optimized pure platinum electrocatalysts.

## Graphical abstract

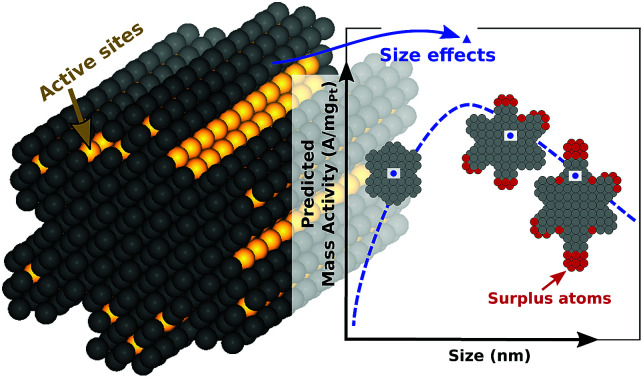

## Introduction

General chemical strategies are currently pursued to accelerate the oxygen reduction reaction (ORR), so as to pave the way for economically viable polymer electrolyte membrane fuel cells.^1^ Recent advances in nanostructure synthesis have greatly aided in the search for nanostructured electrocatalysts which harbor enhanced catalytic mass activities over current commercial Pt/C electrocatalysts.^2^ During the past few years, record mass activities for the ORR have been reported for various nanostructured Pt-alloy electrocatalysts. For instance, PtNi alloys with octahedral nanostructures^3,4^ and PtNi alloy nanoframes^5^ demonstrate superior activities.

Based on the Sabatier principle, which is one of the pillars of modern catalysis, it is widely accepted that the ORR activity is largely controlled by the adsorption energies of its reaction intermediates.^6–8^ More precisely, numerous experimental studies on pure Pt and Pt-alloy model structures unraveled that weakening the *OH adsorption energies by ∼0.1–0.15 eV with respect to Pt(111) gives rise to improved catalytic activities.^9–13^

Furthermore, for a pure metal, the relation between adsorption energies and morphology is provided by structural descriptors such as generalized coordination numbers.^14,15,40^ The generalized coordination number of a given site *i*1
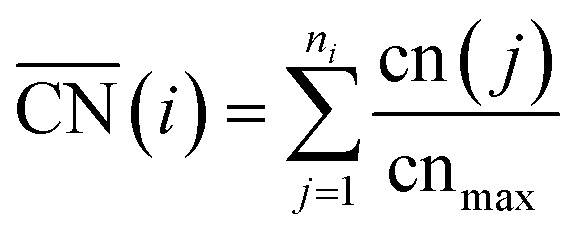
sums up the conventional coordination numbers cn(*j*) of the nearest neighbors *j*, which are *n*_*i*_ nearest neighbors in total. The sum over nearest neighbors is normalized by the bulk atom coordination cn_max_, which is cn_max_ = 12 for bulk sites in the fcc structure. Therefore, 
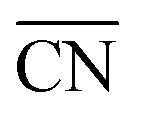
 comprises finite size effects and local site structures up to the second coordination sphere of the active sites. Density Functional Theory (DFT) studies have shown that the adsorption energies of all crucial ORR intermediates, *i.e.* *O, *O_2_, *OH, and *OOH, are linearly related with 
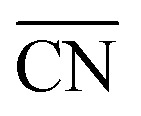
.^13,14^ Generalized coordination numbers also capture strain effects on pure Pt and Pt-alloys, so that strained Pt catalysts approximately follow the same linear trends in *OH and *OOH adsorption energies (for pure Pt)^16^ and *O_2_ adsorption energies (for Pt alloys)^17^ as the unstrained Pt catalysts. This is noteworthy, as a number of recent works have put forward the importance of strain for the ORR.^12,18–21^

Coordination–activity plots based on the Sabatier principle and the DFT-assessed free energies of the intermediates (which are calculated as in ref. 6), show that local Pt site structures with 
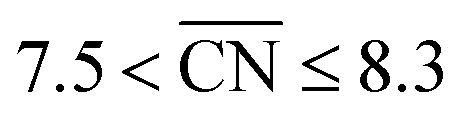
 are expected to yield activity enhancements relative to Pt(111).^2,13,15,16^ Importantly, this range in 
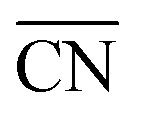
 corresponds to a weakening of ∼0–0.15 eV in *OH binding energy relative to Pt(111), which coincides with the experimental range mentioned above.^9–13^ The effects of the local site structure demonstrate that the interplay between shape and size of nanostructures determines the adsorption energies and, therefore, shape^22,23^ and size^24,25^ engineering are crucial to achieve electrocatalysts with high mass activity.

On certain model Pt stepped surfaces, active sites with 
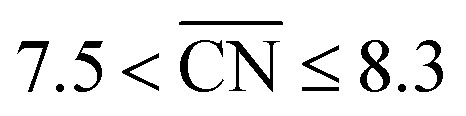
 are arranged in a side-by-side formation.^15^ Furthermore, it is suggested that active sites can be tailored in cavities^13^ and at concavities^15,18,26^ of Pt nanostructures.

From an experimental perspective, the synthesis and characterization of several thousands of different nanostructures is time-consuming and expensive. Thus, fast computational screening of nanostructures can provide experimenters with prospective nanostructures to be synthesized, characterized, and evaluated electrocatalytically. However, in computational studies, elaborating detailed mass activity predictions within feasible computation times is also a rather demanding task. For instance, the concept of 
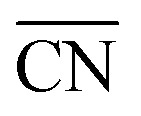
 provides important knowledge of active local site structures, but it does not tell how plenty of those active local site structures can be incorporated into stable nanostructured electrocatalysts with high mass activity.

A recent computational model^27^ allows for the rapid prediction of mass activities of regular nanoparticles, where the structures of Pt electrocatalysts are evaluated at the nanoscale based on 
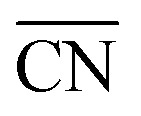
. In that study, mass activities in good agreement with experiments have been computed in units of amperes per milligram.

Herein, we screen shapes and sizes of nanostructures to identify Pt electrocatalysts which feature high mass activity, controllable size distribution, and decent mechanical stability. Since Gielis' Superformula^28^ includes a continuous parameter space to create various kinds of shapes, we propose a generalization of it in three dimensions^29,30^ to design a wide variety of continuous symmetric shapes (see Methods section). Pt nanoparticles are carefully optimized toward highest mass activity by Penalty Attractive and Repulsive Particle Swarm Optimization (PARPSO).^31^ To account for high stability under ORR conditions, low-coordinated sites with cn < 6 are avoided. The enhancement in mass activities of tailored nanostructured electrocatalysts is benchmarked against Tanaka commercial Pt/C electrocatalysts with 0.55 A mg_Pt_^−1^ mass activity.^32^

## Results and discussion

To explore the shapes and sizes of nanostructured Pt electrocatalysts, we will start our analysis with one of the simplest and most common class of shapes, namely sphere-like nanoparticles. The analysis of sphere-like nanoparticles will be followed by the investigation of rod-like nanostructures, which constitute a second, more complex class of shapes. Both types of shapes are generated by two distinct variations of the Superformula. Note that each Superformula shape fulfills either C3 or C4 rotational symmetry with respect to the *z*-axis.

### Sphere-like nanoparticles

**Fig. 1:**
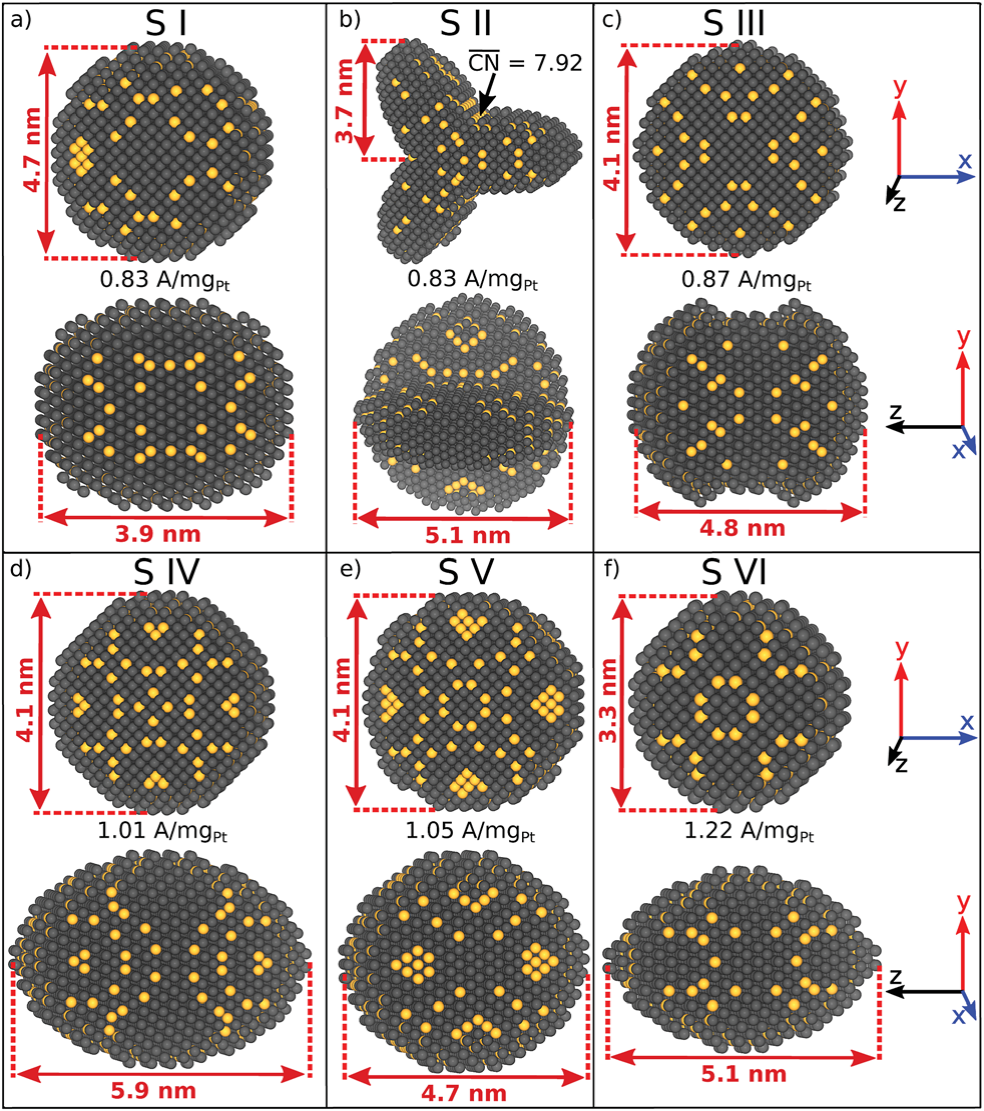
(a–f) Active sphere-like nanoparticles identified by two screenings. Active sites with 
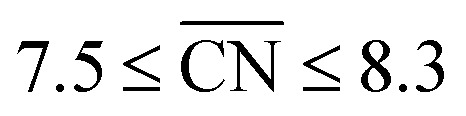
 are highlighted in yellow. Note that, although shape S II has a tripod shape in the *x*–*y* plane, and a sphere-like shape in the *y*–*z* plane, the shape S II originates from the spherical coordinate related Superformula.

Six sphere-like nanoparticles with high predicted mass activities are identified in two different screenings, which are numbered from S I to S VI in Fig. 1. The nanoparticles S I to S V, which are obtained by the first screening (see ESI†), have relatively large sizes from ∼4 nm and mass activities in the range of 0.83 A mg_Pt_^−1^ to 1.05 A mg_Pt_^−1^.

A comparison between S III and S V shows that both nanoparticles have similar sizes, but their different shapes lead to different arrangements of active sites. Both shapes have similar lengths along the *x*-, *y*-, and *z*-axes, but a different surface curvature with respect to the *z*-axis. Consequently, the nanoparticle S V should have 20% larger mass activity compared to S III. This illustrates that nanostructure shapes have significant effects on the catalytic activity for a given size.

Lowering the nanoparticle size to 3.3 nm along the *x*- and *y*-axes (see Fig. 1f) renders nanoparticle S VI, which is obtained by the second screening. The shape S VI has an increased predicted mass activity of 1.22 A mg_Pt_^−1^, corresponding to a ∼2.2-fold enhancement over Tanaka commercial Pt/C electrocatalysts.^32^

**Fig. 2:**
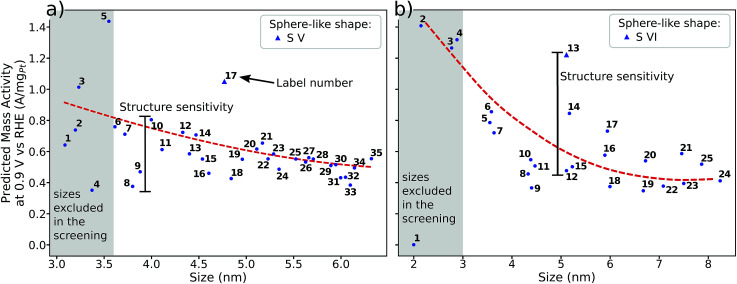
Size effects on the predicted mass activity for the sphere-like shapes (a) S V and (b) S VI. All nanoparticles in (a) and (b) are illustrated in the ESI.† The red dashed curves are provided as a guide to the eye to indicate the overall mass activity trend. Sizes below ∼3.6 nm in (a) and ∼3 nm in (b) (see grey shaded areas) are excluded in the screenings to focus on intermediate sizes. Note that panels (a) and (b) have the same mass activity scale.

Albeit the nanoparticles S V and S VI are the most mass-active shapes and sizes identified in the two associated screenings, their mass activities depend sensitively on the size. To focus on intermediate sizes, a size limit of ∼3.6 nm is used in the screenings (see ESI†). Above this size limit, Fig. 2a reveals that the shape S V is optimal in size, so that it should be 30% more mass-active than other electrocatalysts of similar sizes. Furthermore, the structure sensitivity increases toward small nanostructure sizes, in line with ref. 33. This observation is rationalized considering that slight changes in the structure of the active sites have stronger effects on the mass activity for small-sized and lightweight nanostructures. In contrast, for larger nanostructures, the mass activities follow more closely the overall trend.

**Fig. 3:**
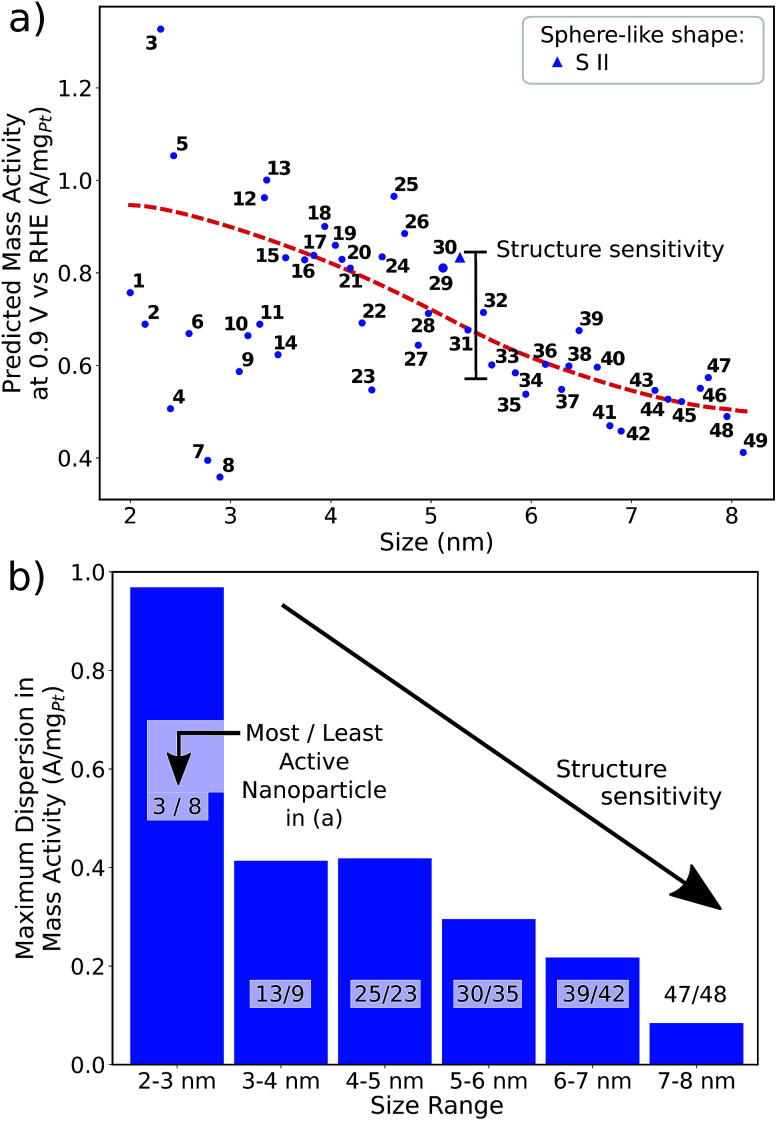
(a) Size effects on the predicted mass activity for the sphere-like shape S II. The red dashed curve is provided as a guide to the eye to indicate the overall mass activity trend. (b) The maximum dispersion in mass activity, *i.e.* the mass activity difference between the most active and the least active nanoparticles, is depicted for several size ranges.

Nanoparticle S VI exhibits similar size effects. Above the size limit of ∼3 nm included in the screening, the mass activity of S VI is optimized with respect to the particle size. The enhanced activity of shape S VI originates from a high distribution of active sites near the optimal value 
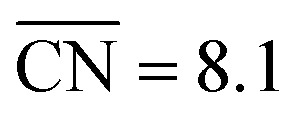
, while less active Pt(111) sites with 
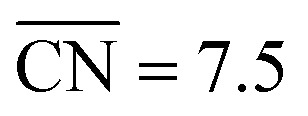
 dominate on electrocatalyst 12 of similar size (see Fig. S8†). Below the size limit of ∼3 nm, higher mass activities up to 1.38 A mg_Pt_^−1^ are detected for electrocatalysts 2, 3, and 4. Unlike shapes S V and S VI, the shape S II is ranked 31st in the mass activity in the associated screening. Since the optimization algorithm focuses on the optimization of the most mass-active nanostructures, lowering the size of S II may improve the mass activities, as shown in Fig. 3. Averaging over the electrocatalysts 12–30 yields a mass activity of 0.81 A mg_Pt_^−1^ with a mean nanostructure size of ∼4.31 nm and a relatively large size distribution of 0.98 nm.

In line with the above discussion, the structure sensitivity of shape S II decreases toward larger nanoparticle sizes. Fig. 3b shows that the dispersion in mass activity between the most active and least active nanoparticles is 1 A mg_Pt_^−1^ at small sizes in the 2–3 nm range. Toward larger sizes, the dispersion is reduced by 90%, where 0.1 A mg_Pt_^−1^ is observed in the 7–8 nm size range.

Shape S II provides important information on how active local site structures with 
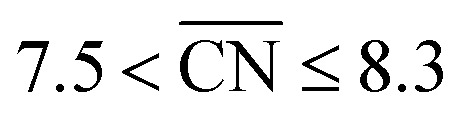
 can be created in nanostructured electrocatalysts. Five active sites up to 
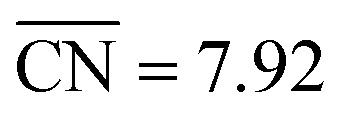
 are lined up side by side at the 120° kink as shown in Fig. 1b. This observation demonstrates that optimal local site structures near 
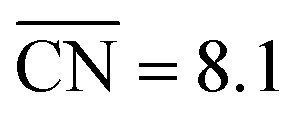
 can be tailored by means of concave kinks. However, the convexity along the 120° kink in S II leads to downgraded 
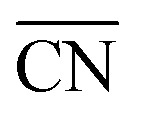
, and therefore less active sites at the edges of the nanoparticle. This problem is resolved in the rod-like nanostructures discussed below.

### Rod-like nanostructures

**Fig. 4:**
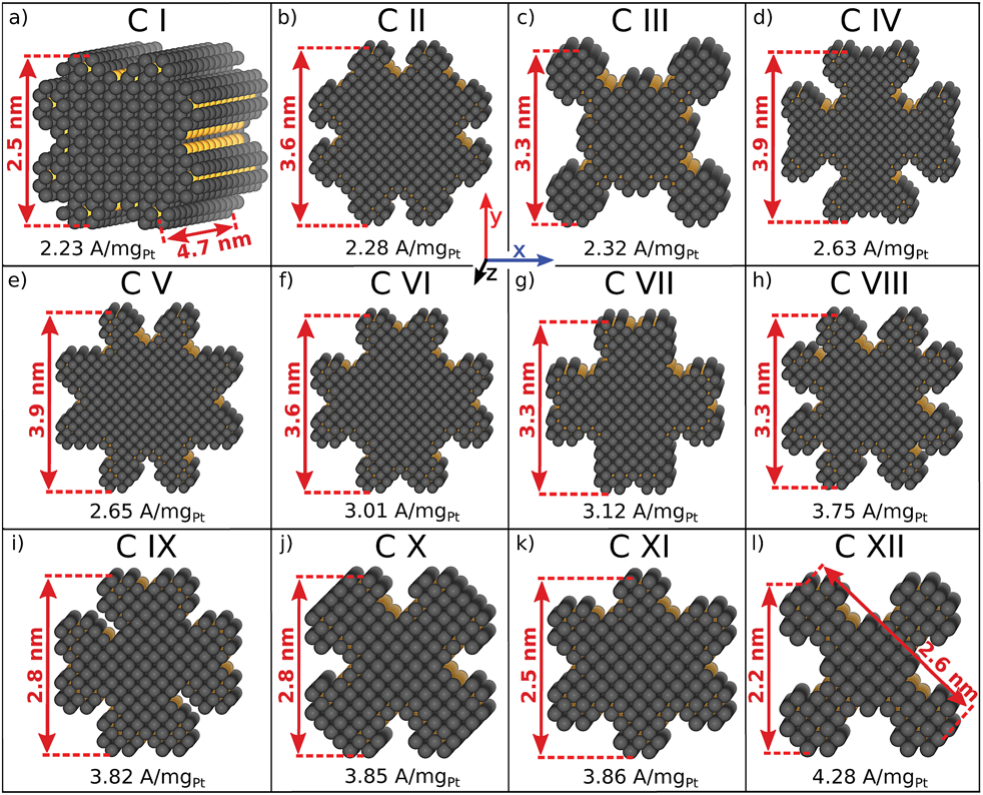
(a–l) Active nanostructured electrocatalysts with rod-like shapes identified by the screenings. Active sites with 
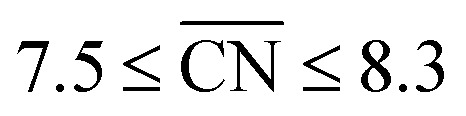
, which are taken into account for mass activity prediction, are highlighted in yellow.

The rod-like nanostructures presented in this study feature rectilinear kinks without convexities, so that active local site structures are periodically extended over the nanostructure length along the *z*-axis, as shown in Fig. 4a and S1.† We present 12 rod-like nanostructures numbered from C I to C XII in Fig. 4, which are obtained from three different screenings. The length along the *z*-axis is fixed to ∼4.7 nm as illustrated in Fig. 4a. To account for arbitrary length in this study, sites at the outer edge along the *z*-axis are excluded for the prediction of mass activities (see Fig. S1†).

The nanostructures have lengths from 2.2 nm to 3.9 nm along the *x*- and *y*-axes. Nanostructures with relatively large lengths from 3.3 nm in Fig. 4b–h harbor mass activities up to 3.75 A mg_Pt_^−1^. Smaller nanostructures in Fig. 4i-l reach mass activities up to 4.28 A mg_Pt_^−1^, corresponding to a ∼7.8-fold enhancement in mass activity over Tanaka commercial Pt/C electrocatalysts.^32^ Thus, the rod-like nanostructures in Fig. 4 are over 3 times more active than the sphere-like nanoparticles in Fig. 1.

Comparing C III and C XII, both nanostructures have similar shapes whereas the sizes differ by almost 1 nm. The smaller nanostructure C XII has almost twofold mass activity over the larger nanostructure C III. In contrast, the nanostructures C I and C XI have similar sizes, but their different shapes result in markedly different mass activities. Again, this suggests that the interplay between nanostructure shapes and sizes is crucial to find nanocatalysts with high mass activities.

**Fig. 5:**
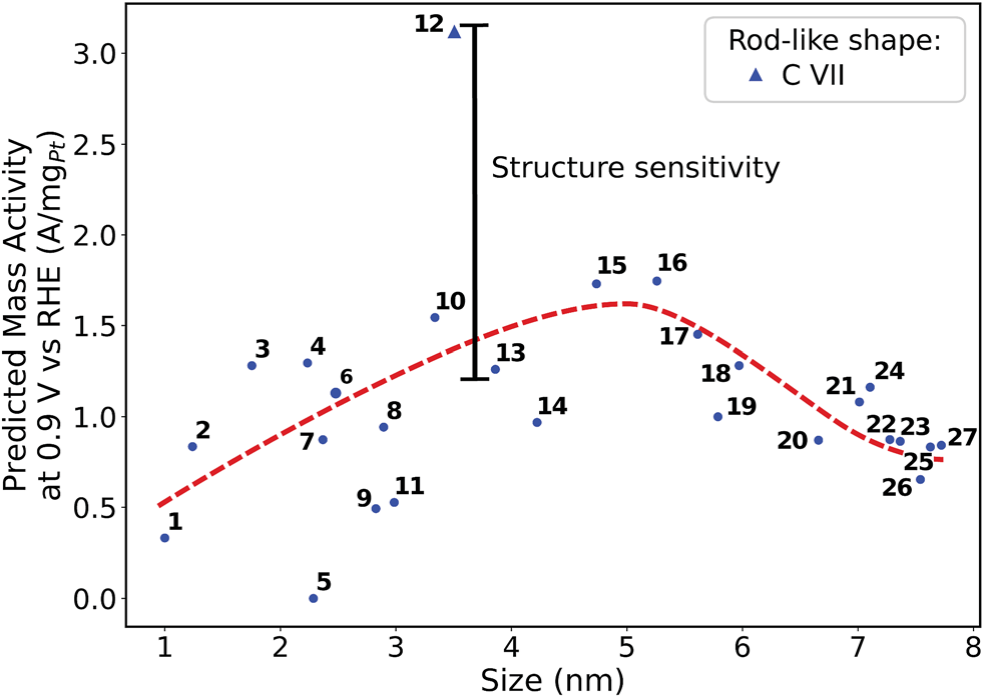
Size effects on the predicted mass activity for the rod-like shape C VII. The red dashed curve is provided as a guide to the eye to indicate the overall mass activity trend.

**Fig. 6:**
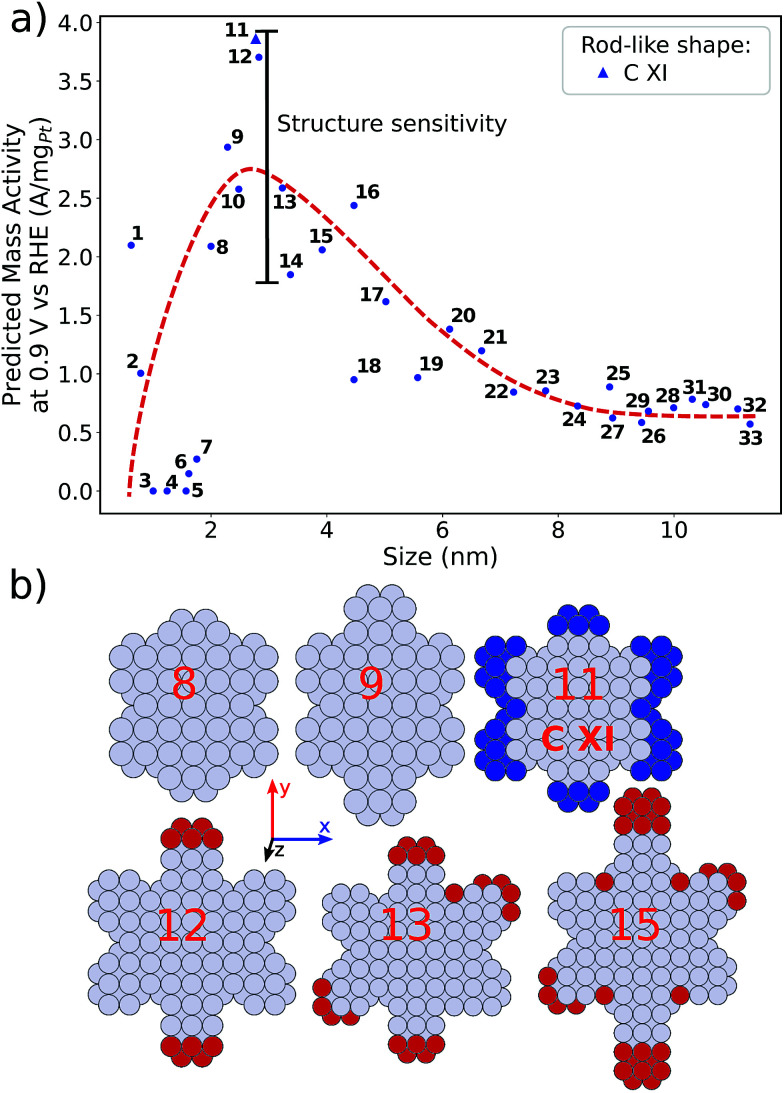
(a) Size effects on the predicted mass activity for the rod-like shape C XI. The red dashed curve is provided as a guide to the eye to indicate the overall mass activity trend. (b) Electrocatalysts 8, 9, 11–13, and 15 are presented. Shortened nanostructure legs in electrocatalyst 8, compared to C XI, are highlighted by blue atoms. Enlarged nanostructure legs of electrocatalysts 12, 13, and 15, compared to C XI, are colored in red.

**Fig. 7:**
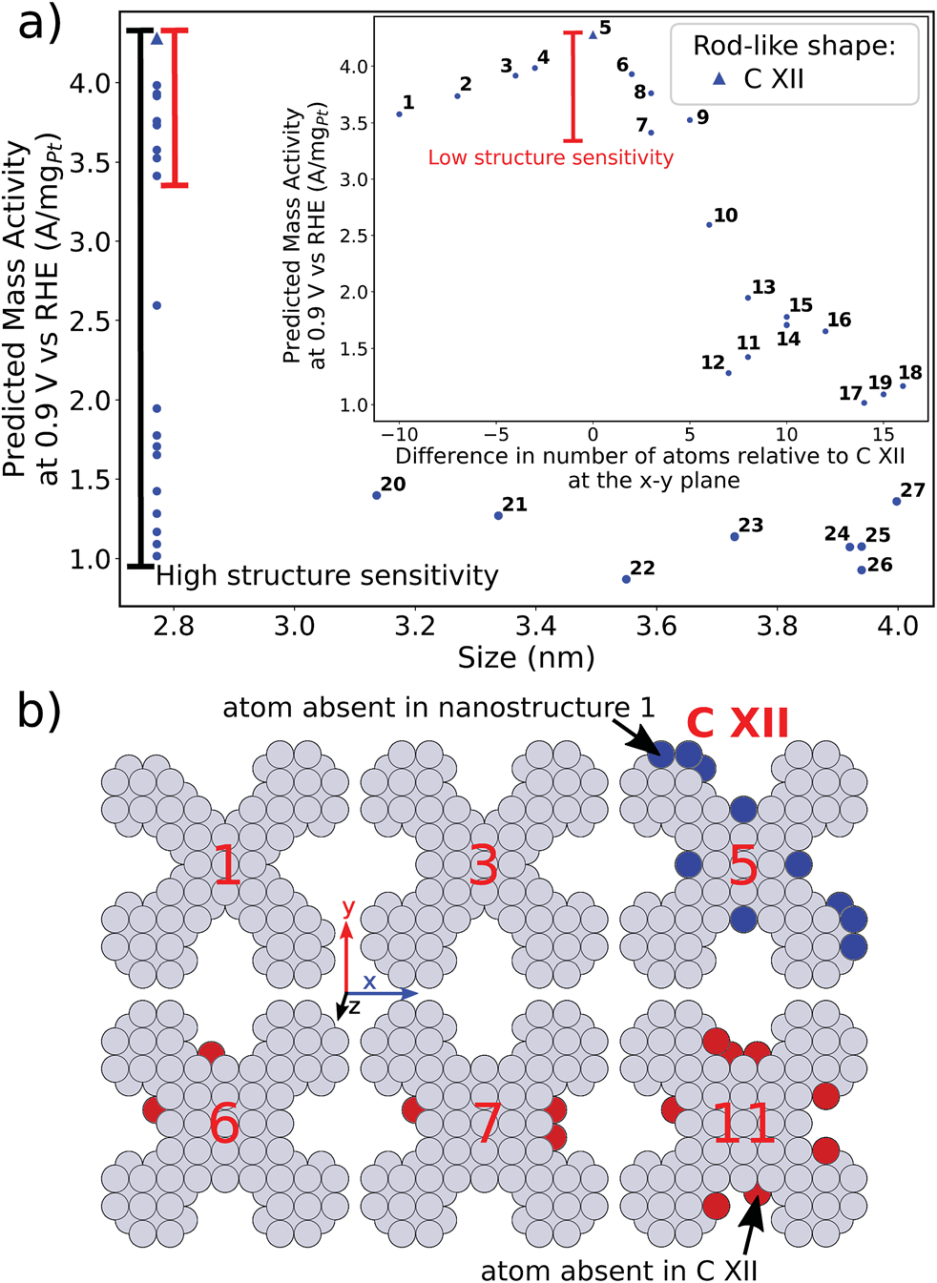
(a) Size effects on the predicted mass activity for the rod-like shape C XII. For electrocatalysts 1–18, the mass activities *versus* the difference in the number of atoms relative to C XII at the *x*–*y* plane are shown in the inset. The structure sensitivity between electrocatalysts 1–18, which have similar overall sizes, is illustrated by the black horizontal bar. The red horizontal bar shows the relatively small structure sensitivity between electrocatalysts 1–9 which differ in the number of additional atoms. (b) Electrocatalysts 1, 3, 5–7, and 11 are presented. Additional atoms, which are absent in electrocatalyst in C XII, are highlighted in red. Atoms in C XII, which are absent in electrocatalyst 1, are highlighted in blue.

Size effects are studied for the most mass-active electrocatalysts in the associated screenings, *i.e.* C VII, C XI and C XII (for the size measure, please see the Methods section on Nanostructure size effects). As depicted in Fig. 5, the relatively large nanostructure C VII is optimal in size, whereas small changes in the local site structures give rise to considerably downgraded mass activities by *ca.* 50% for electrocatalysts 10 and 13. The large difference in mass activity can be rationalized from the 
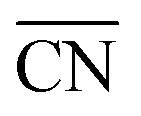
 distribution (provided in the ESI†). For instance, electrocatalyst 10 has only half as many active sites compared to the optimized shape C VII of slightly larger size. Thus, experimental activity measurements on C VII would strongly depend on the size distribution at the atomic scale. The smaller and more mass-active nanostructure C XI is also optimal in size, and small size variations have large effects on the mass activity, as well. This is depicted in Fig. 6. In particular, the electrocatalysts 8–15 in Fig. 6 feature the same nanostructure centers, but the lengths of the legs are different. Shortening the legs of C XI (see electrocatalyst 8) and elongating them (see electrocatalysts 12, 13, and 15) downgrade the mass activity. Nonetheless, a high average mass activity of 2.71 A mg_Pt_^−1^ is predicted at mean nanostructure size (2.96 ± 0.96) nm for electrocatalysts 8–15. This constitutes a 4.9-fold mass activity increase over Tanaka commercial Pt/C electrocatalysts. Size effects on the most mass-active nanostructure C XII are presented in Fig. 7a. Although electrocatalysts 1–18 are all 2.6 nm in size, the associated mass activities are widely scattered. A detailed evaluation of electrocatalysts 1–18 reveals that they have different local site structures at concave kinks. Plotting the mass activities *versus* the number of atoms relative to nanostructure C XII (see inset in Fig. 7a) uncovers two groups of nanostructures. Electrocatalysts 1–9 have high mass activities of 3.41–4.27 A mg_Pt_^−1^ whereas the electrocatalysts 10–18 have significantly lower mass activities in the range of 1.02–2.59 A mg_Pt_^−1^.

As illustrated in Fig. 7b, the difference in the number of atoms relative to C XII, which are located at the *x*–*y* plane and highlighted in red, increases from 2 to 8 on electrocatalysts 6–11. In the case of only few additional atoms, as for electrocatalysts 6 and 7, the mass activities are still on a high level near 3.41 A mg_Pt_^−1^ and above. Absence of several atoms (highlighted in blue) on electrocatalysts 1–4 in fact keeps the mass activity above 3.5 A mg_Pt_^−1^. In contrast, more than *ca.* 5 additional atoms results in considerably downgraded mass activity as illustrated for electrocatalyst 11.

Thus, realizing C XII with diagonal size of 2.6 nm (see Fig. 4) and few additional atoms would enable high mass activities of ∼3.79 A mg_Pt_^−1^, corresponding to the average mass activity of electrocatalysts 1–9.

## Experimental perspective

**Fig. 8:**
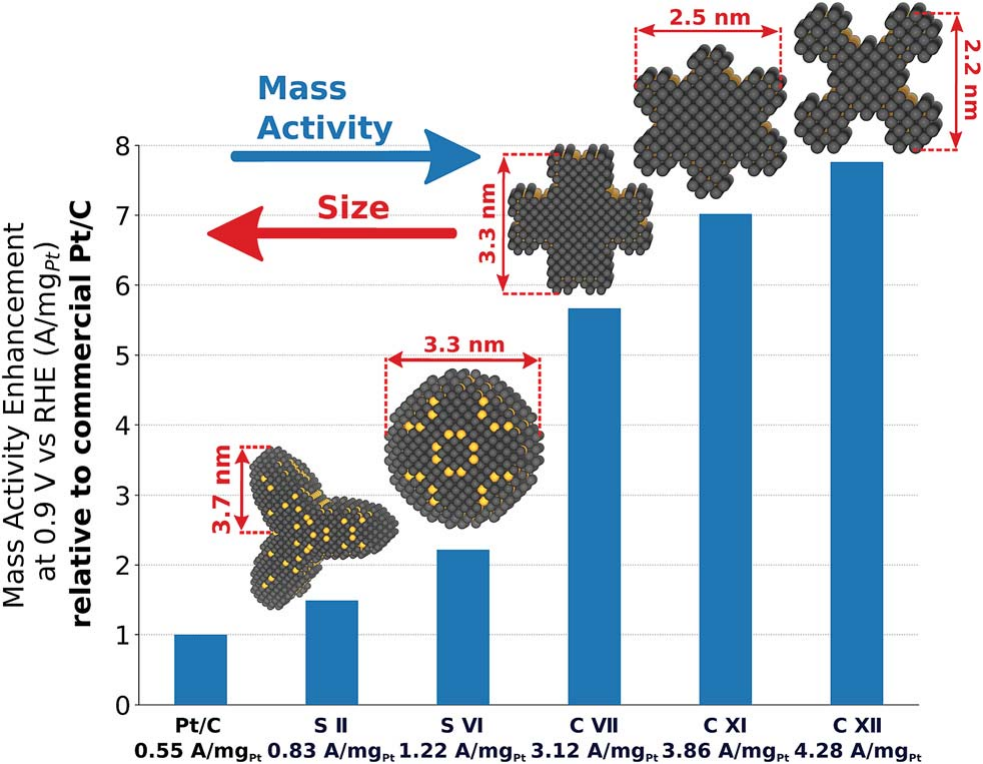
Mass activity enhancement of S II, S VI, C VII, C XI, and C XII over Tanaka commercial Pt/C electrocatalysts.^32^

From an experimental perspective, it is useful to summarize the mass activity enhancement of tailored nano-electrocatalysts compared to Tanaka commercial Pt/C electrocatalysts, as in Fig. 8. The sphere-like nanoparticle S II harbors relatively small mass activity enhancement of ∼150% over Tanaka commercial Pt/C electrocatalysts, but low structure sensitivity likely allows for straightforward experimental synthesis.

The shape S VI achieves the highest mass activity enhancement among all sphere-like shapes investigated here. However, large structure sensitivity for similar sizes would necessitate narrow size distributions to reach in experiments the predicted mass activity of 1.22 A mg_Pt_^−1^.

Because of active and abundant local site structures at concave nanostructure kinks, rod-like nanostructures are predicted to have remarkably high mass activities. Nonetheless, C VII exhibits large structure sensitivity, which may downgrade the high mass activity. In contrast, the two most mass-active electrocatalysts, namely C XI and C XII, feature controllable structure sensitivity and mass activity enhancements of ∼702% and ∼776% over Tanaka commercial Pt/C electrocatalysts. These structures are representative in the sense that high average mass activities from 2.7 A mg_Pt_^−1^ to 3.7 A mg_Pt_^−1^ are maintained upon possible structural variations.

It is noteworthy that similar nanocatalyst shapes of Pt-alloys have been presented before in experiments^34,35^ and in theoretical studies.^26^ The pure Pt nanocatalysts proposed in this study may likely be synthesized using templates, for which metal–organic frameworks are suitable.^36^ High-resolution transmission electron microscopy could be used to resolve their structures at the subnanoscale.^37^

Beside high mass activity and controllable structure sensitivity, electrocatalyst stability is a critical aspect in fuel cell environments. To account for high stability under ORR conditions, low-coordinated sites with cn < 6 are avoided, except for sites placed at the outer edge in *z*-direction of rod-like nanostructures (see Fig. S1†). Importantly, Kibsgaard *et al.* have investigated the stability of Pt mesostructures with respect to the site coordination.^38^ The fraction of edge sites and corner sites in such Pt mesostructures is determined to be 11%. The coordination of such sites, which is given in range of cn = 5 to cn = 7, is similar to the minimal coordination cn = 6 in our study. However, it has been shown experimentally that those Pt mesostructures feature increased stability over ETEK commercial Pt/C electrocatalysts. Hence, this experimental insight strongly suggests that the condition cn ≥ 6 for the sites of any electrocatalyst in our study is an appropriate constraint to achieve stable tailored nanostructured electrocatalysts. We further note that the above-mentioned stellated shapes,^34,35^ which contain Pt-rich shells and Ag-rich cores, are an indication for appropriate intrinsic stability of such shapes. Ref. 18 (and references therein) also shows that a variety of Pt-based concave nanoparticles can be synthesized experimentally, which are ORR active and fairly stable.

## Conclusions

In this article, we have proposed a theoretical approach to identify pure Pt electrocatalysts with high ORR mass activities, appropriate mechanical stability and controllable structure sensitivity through careful variations in their sizes and shapes. The generalization of Gielis' Superformula in three dimensions is used to design a wide variety of symmetric shapes with tunable size, which are optimized toward highest mass activity by the particle swarm optimizer PARPSO.

Our analysis of sphere-like nanoparticles predicts mass activities up to 1.22 A mg_Pt_^−1^. Moreover, rod-like nanostructures are found to be over 3 times more active than sphere-like nanoparticles. For rod-like nanostructures, we predict mass activities up to 4.28 A mg_Pt_^−1^, corresponding to a ∼7.8-fold enhancement over Tanaka commercial Pt/C electrocatalysts. Such high activities originate from numerous active sites located at concave kinks, which are tailored toward optimal coordination near 
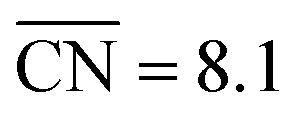
. Upon small size changes, the most active rod-like nanostructures retain high mass activities in some cases above 3.41 A mg_Pt_^−1^. The comparison with recent experimental stability studies strongly suggests that the nanocatalysts proposed in this article are at least as stable as commercial Pt/C electrocatalysts.

In sum, the high predicted mass activities of pure Pt electrocatalysts from this study show great potential to reduce the Pt loading in PEMFCs. The chemical routes for synthesis, which are obtained from the size effect studies, may foster and support upcoming experimental works on pure Pt electrocatalysts.

## Computational methods

### Prediction of mass activities

A recently developed computational model performs rapid prediction of mass activities of nanostructured Pt electrocatalysts in absolute units of amperes per milligram.^27^ Within that computational model, catalytic activities are predicted upon evaluation of local site structures described by generalized coordination numbers 
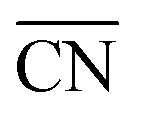
.^13–15,40^ Particularly, the model involves

• a linear relation between 
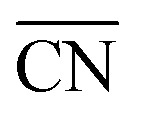
 and *OH binding energies with respect to the gas phase from DFT studies and

• experimental ORR activities *versus* *OH binding energies given relatively to Pt(111), which are measured on pure Pt and Pt-alloy electrocatalysts.

The linear DFT relation is used to map the experimental *OH binding energies onto 
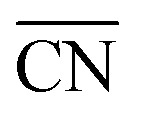
, which yields the fitted volcano-shaped activity trend2
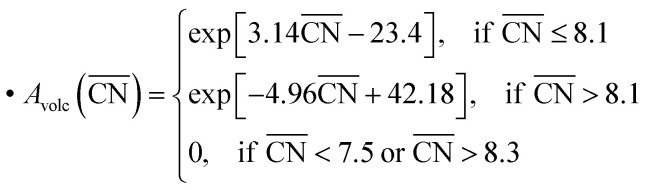
between experimental activities *A*_volc_ relative to Pt(111) and 
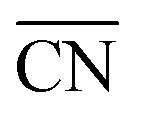
. The highest activity contribution with respect to Pt(111) is given by 
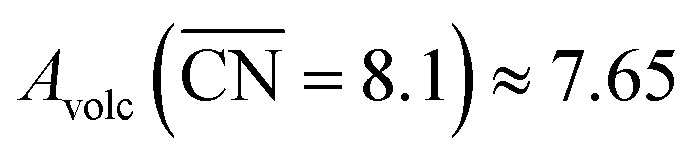
, where 
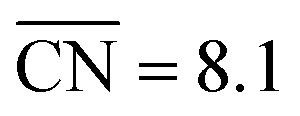
 corresponds to a weakening of 0.115 eV in *OH binding energy relative to Pt(111). Since enhanced activities over Pt(111) are expected for sites with 
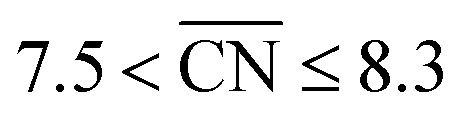
 from the Sabatier analysis,^13,15^ activity contributions from sites with 
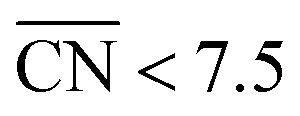
 and 
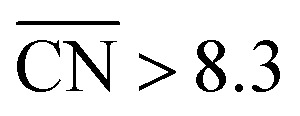
 are neglected in *A*_volc_ (see also ref. 27 for a more detailed discussion). Hence, the edges of the volcano-shaped activity trend are given by *A*_volc_ (7.5) ≈ 1.16 and *A*_volc_ (8.3) ≈ 2.75.

Employing the Pt atomic mass *m*_Pt_ = 195.084 *u*, the site density on the Pt(111) surface *d*_Pt(111)_ = 1.503 × 10^15^ cm^−2^ and the specific activity *j*_Pt(111)_ = 2 mA cm^−2^ of Pt(111),^13^ mass activities are calculated by summing over activity contributions as3
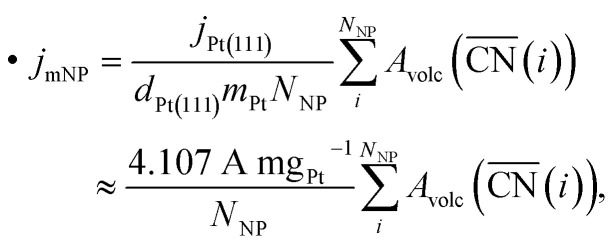
where *N*_NP_ denotes the number of atoms comprised by the nanoparticle. Thus, mass activities are predicted in absolute units of A mg_Pt_^−1^ upon geometrical evaluation of local site structures, which are described by 
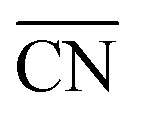
.

### Tailoring nanostructured Pt electrocatalysts

Gielis' Superformula provides a wide variety of shapes.^28^ A three-dimensional version of the Superformula has been applied on nanophotonic^30^ and dielectric^29^ materials. We introduce a generalization of the Superformula in three dimensions which fulfills C3 or C4 rotational symmetries with respect to the *z*-axis. All Superformula shapes are C2 symmetric around the *x*- and *y*-axes.

Discrete surface meshes are constructed from shapes which are generated by the generalized Superformula. Surface meshes are used to cut the shape out of the Pt bulk. Note that the Pt fcc bulk structure is itself C4 symmetric such that C3 symmetric Superformula shapes do not generally yield C3 symmetric nanostructures. Furthermore, the discrete surface mesh points are not necessarily distributed in a symmetric arrangement which may lead to small symmetry deviations in the nanostructures. This feature is exploited to study structure sensitivity arising from asymmetrically added atoms.

Beyond that, low-coordinated sites with cn < 6 are strictly avoided, except for sites placed at the outer edges in *z*-direction of rod-like nanostructures (see ESI†). We use the Atomic Simulation Environment (ASE)^39^ for calculations and visualization of nanostructures.

The generalized Superformula in three dimensions is given by4
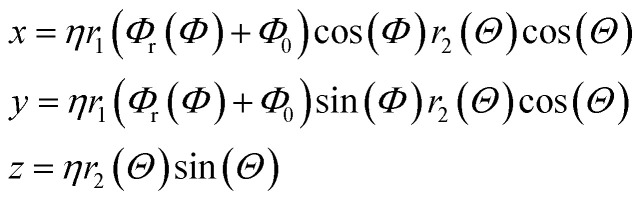
where5
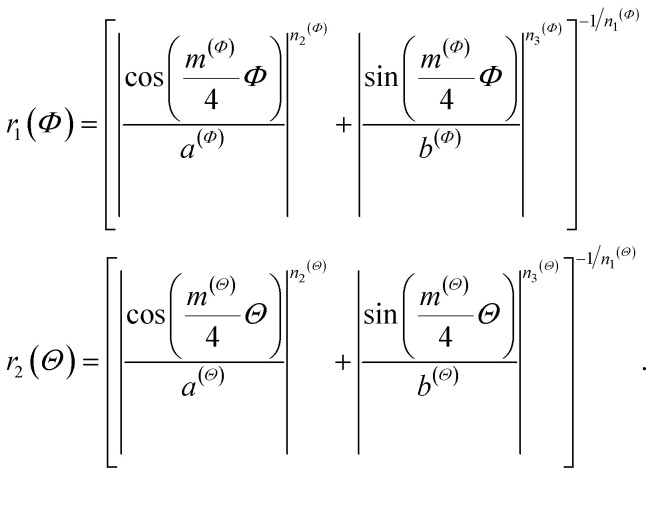


Since eqn (4) is related to spherical coordinates, nanostructures obtained from eqn (4) are referred to as sphere-like nanoparticles. The Superformula in eqn (4) comprises fourteen distinct parameters. Each six of them, *i.e. m*^(*Φ*)^, *n*_1_^(*Φ*)^, *n*_2_^(*Φ*)^, *n*_3_^(*Φ*)^, *a*^(*Φ*)^, *b*^(*Φ*)^ and *m*^(*Θ*)^, *n*_1_^(*Θ*)^, *n*_2_^(*Θ*)^, *n*_3_^(*Θ*)^, *a*^(*Θ*)^, *b*^(*Θ*)^ are associated with the azimuthal angle *Φ* and the altitude angle *Θ*, respectively. The size parameter *η* defines the size of the shape. The three dimensional space is spanned by *Φ∈*[−π, π] and *Θ∈*[−π/2, π/2].

Since rotational symmetries may strongly facilitate experimental synthesis, we construct Superformula shapes which fulfill C3 and C4 symmetries. The functions6
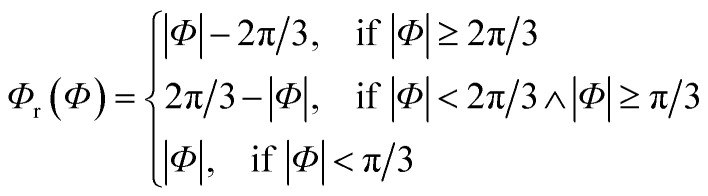
for C3 symmetry and7
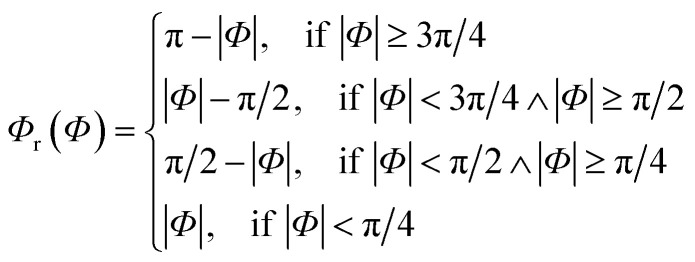
for C4 symmetry map the full domain of the variable *Φ∈*[−π, π] onto the subset *Φ*_r_(*Φ*)∈[0, *c*] where we define *c* = π/3 for C3 symmetry and *c* = π/4 for C4 symmetry. Furthermore, arbitrary choice of the parameter *Φ*_0_ in range of [0, π], which is included in eqn (4), allows to select any section of the full domain *Φ∈*[−π, π] of the Superformula shape. Therefore, the generalized Superformula in eqn (4) comprises 14 parameters in total. Note that choosing the identity function *Φ*_r_(*Φ*) = *Φ* and *Φ*_0_ = 0 reduces the generalized Superformula in eqn (4) to the ordinary Superformula in three dimensions which is used in ref. 29 and 30.

In addition to sphere-like nanoparticles, we investigate rod-like nanostructures generated by the Superformula8
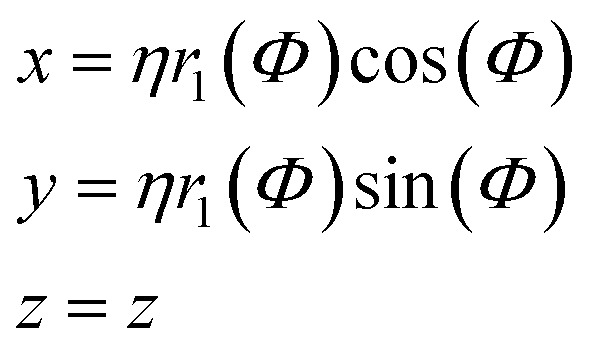
which is related to cylindrical coordinates. Such shapes can be arbitrarily extended in the *z*-direction, whereas the range *z∈*[0, *z*_max_] with *z*_max_ = 2.5 nm is kept constant in this study. To consider arbitrary extensions of the rod-like nanostructures, finite-size effects in the *z*-direction are excluded for activity predictions (see Fig. S1†). The Superformula in eqn (8) comprises 8 parameters in total.

For further details on the examined nanostructures and the associated Superformula parameters, we refer the reader to the ESI.†

### Nanostructure screening and optimization

To identify the most mass-active nanostructures in the screenings of tailored nanostructures, we use Penalty Attractive and Repulsive Particle Swarm Optimization (PARPSO).^31^ Nanostructures are optimized toward highest mass activity, where the optimization scheme includes four major steps:

(1) *N* = 50 parameter ensembles are initialized randomly. Note that each parameter ensemble comprises 14 parameters (for sphere-like nanoparticles) or 8 parameters (for rod-like nanostructures), which are involved in the two Superformulas in eqn (4) and (8).

(2) *N* = 50 nanostructures are constructed from the Superformula in eqn (4) for sphere-like nanoparticles or eqn (8) for rod-like nanostructures.

(3) Mass activities of all *N* = 50 nanostructures are determined by eqn (3).

(4) PARPSO slightly modifies the single parameters of all *N* nanoparticle ensembles toward optimized mass activity. This step is performed under the influence of random fluctuations and previous information about the mass activity, for which the mathematical details are presented below. Unless the maximum iteration *k*_max_ is reached, the algorithm continues with step 2.

For each screening, *k*_max_ = 604 to *k*_max_ = 800 iterative optimization steps are performed. Specific details about all screenings, including the optimized parameter ensembles, are provided in the ESI.† Thus, up to 40 000 nanostructures are evaluated within one screening. Using parallel computing in each iterative step and rapid prediction of mass activities,^27^ one screening is performed within few days of computation time.

PARPSO considers three overall aspects to optimize nanostructures parameter ensembles toward highest mass activity in step 4. Each parameter ensemble *x*_*j*_^*k*^ at iterative step *k* is optimized with respect to a tradeoff between the best parameters *p*_best,*j*_^*k*^ of each parameter ensemble and the overall best parameters *g*_best_^*k*^ out of all parameter ensembles. The variable *d*^*k*^, taking values 1 or −1, balances the search near *p*_best,*j*_^*k*^ against the search near *g*_best_^*k*^. In addition, PARPSO involves a penalty factor *w*_2_ to repulse each parameter ensemble from the worst parameters *g*_worst_^*k*^ out of all parameter ensembles.

In each iterative step *k*, each parameter ensemble is updated as *x*_*j*_^*k*+1^ = *x*_*j*_^*k*^ + *V*_*j*_^*k*+1^ with

9 *V*_*j*_^*k*+1^ = *w*_1_^*k*^*V*_*j*_^*k*^ + *d*^*k*+1^[*c*_1_*r*_1_^*k*^(*p*_best,*j*_^*k*^ − *x*_*j*_^*k*^) + *c*_2_*r*_2_^*k*^(*g*_best_^*k*^ − *x*_*j*_^*k*^)] − *w*_2_^*k*^*c*_3_*r*_3_^*k*^(*g*_worst_^*k*^ − *x*_*j*_^*k*^).

The variable *d*^*k*+1^ is given by10
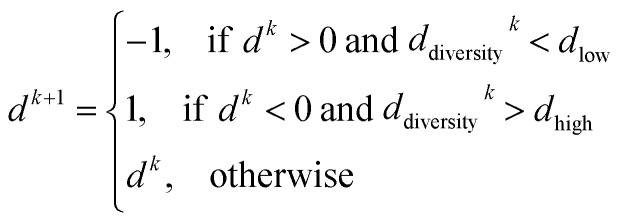
with thresholds chosen as *d*_low_ = 0.4 and *d*_high_ = 0.6 using the diversity function11
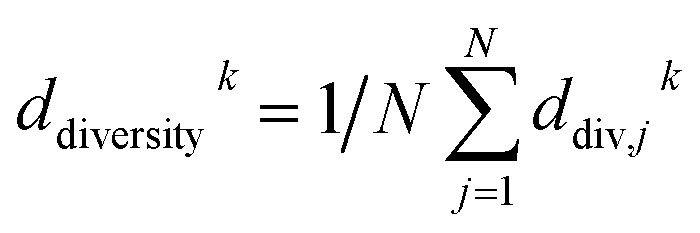
and12
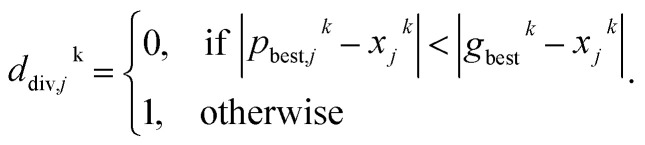


If most parameter ensembles are located near *p*_best,*j*_^*k*^ (*g*_best_^*k*^), the diversity *d*_diversity_^*k*^ will take values near zero (one). Furthermore, the penalty factor *w*_2_^*k*^ is given by13
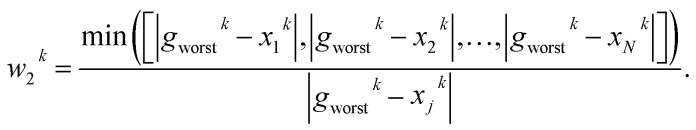


The prefactor14
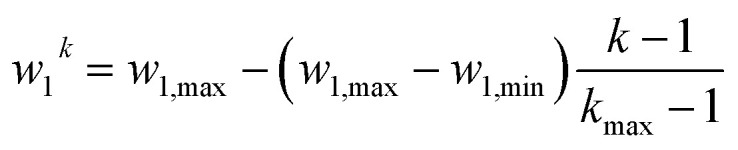
decreases iteratively from *w*_1,max_ = 0.9 to *w*_1,min_ = 0.1 where the iterative steps are counted from *k* = 1 to *k* = *k*_max_. Three uniformly distributed random numbers *r*_1_, *r*_2_, and *r*_3_ are generated in each iterative step in the range of [0, 1]. The three overall constants are defined as *c*_1_ = 1, *c*_2_ = 2, and *c*_3_ = 0.4.

In this way, our high-throughput screening is highly focused on the search for the most mass-active electrocatalyst, though electrocatalysts with slightly lower mass activity are also taken into account. *Ca.* 175 000 shapes are screened in total.

### Nanostructure size effects

Changes in the nanostructure size upon varying the size parameter *η* in the Superformula are analyzed in this study. For sphere-like nanoparticles, the size measure corresponds to the largest distance between any two atoms of the nanostructure. For rod-like nanostructures, the size measure is defined as the largest distance between any two atoms in the *x*–*y* plane of the nanostructure. As discussed above, all nanostructures are obtained by cutting the Superformula shape out of the Pt fcc bulk. Low-coordinated sites with cn < 6 are avoided, except for sites located at the outer edges in the *z*-direction of rod-like nanostructures (see ESI†).

## Conflicts of interest

There are no conflicts to declare.

## Supplementary Material

NA-001-C9NA00252A-s001
