# Supplementary material for: Fast identification of optimal pure platinum nanoparticle shapes and sizes for efficient oxygen electroreduction
Source: Nanoscale Adv. 2019 Jun 3;1(8):2901–9. doi: 10.1039/c9na00252a (PMC9418472; doi:10.1039/c9na00252a)
Supplement: NA-001-C9NA00252A-s001 [file NA-001-C9NA00252A-s001.pdf]

Electronic Supplementary Material (ESI) for Nanoscale Advances.
This journal is © The Royal Society of Chemistry 2019

Supporting Information

Fast Identification of Optimal Pure Platinum Nanoparticle Shapes and Sizes for Efficient Oxygen Electroreduction

Marlon Rück,[†] Aliaksandr Bandarenka,[‡] Federico Calle-Vallejo,[¶] and Alessio Gagliardi[*,†]

†*Department of Electrical and Computer Engineering, Technical University Of Munich, 80333 München, Germany*

‡*Physics Department, Technical University Of Munich, 85748 Garching, Germany*

¶*Department of Materials Science and Physical Chemistry, Institute of Theoretical and Computational Chemistry (IQTC), University of Barcelona, 08028 Barcelona, Spain*

E-mail: alessio.gagliardi@tum.de

Rod-like Nanostuctures

For rod-like nanostructures, active local site structures are periodically extended over the nanostructure length along the z-axis, as shown in Figure S1. To consider arbitrary length of the nanostructure along the z-axis, sites located at the two outer atomic layers (colored in light gray in Figure S1) are neglected for the prediction of mass activity.

To account for high nanostructure stability under ORR conditions, low-coordinated sites with conventional coordination number $cn < 6$ are avoided, except for those sites which are located at the two outer atomic layers (colored in light gray in Figure S1) of rod-like nanostructures. For instance, low-coordinated sites with $cn = 4$ and $cn = 5$ are present at the outer edge of the nanostructure C I, as depicted in Figure S1.

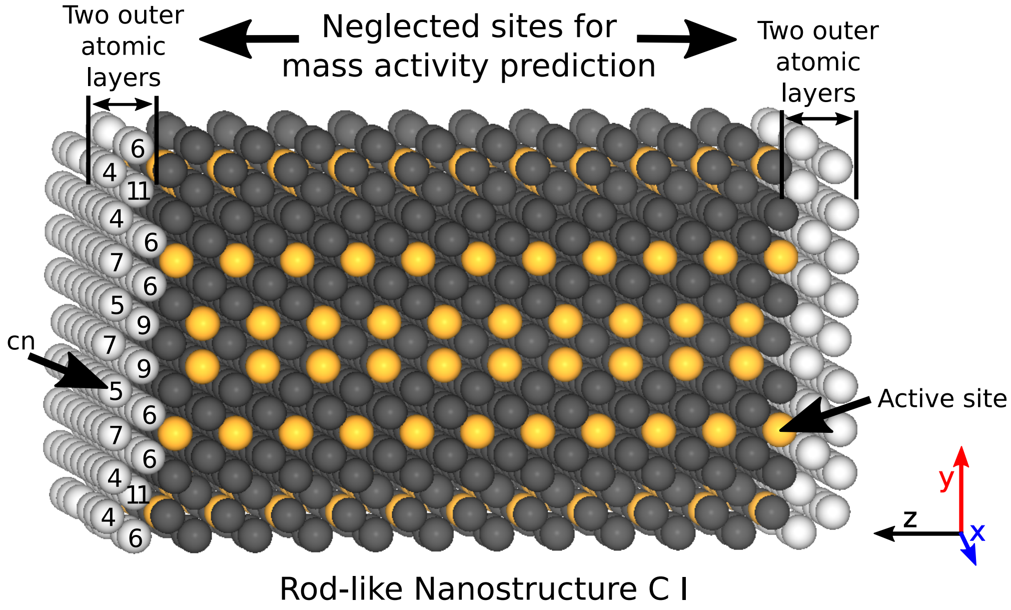

Figure S1: Rod-like nanostructure C I. Active sites with $7.5 \leq \overline{CN} \leq 8.3$ are highlighted in yellow. The two outer atomic layers along the z-axis, which are neglected for the prediction of mass activity, are colored in light gray. The conventional coordiantion number (cn) is displayed for some sites of the two outer atomic layers along the z-axis.

Details of Screenings and Parameter Ensembles

Superformula Parameter Spaces

In this section, the chosen Superformula parameter spaces and the details on nanostructure size constraints are provided. For the screenings 2-5 (see details below), the Superformula parameter space is chosen as

$$\begin{gathered} 0.15 \leq m^{(\Phi)} \leq 12 \quad , \quad 0.15 \leq m^{(\Theta)} \leq 6 \\ 0.15 \leq n_1^x \leq 12 \quad , \quad 0.15 \leq n_2^x \leq 12 \quad , \quad 0.15 \leq n_3^x \leq 12 \\ 0.15 \leq a^x \leq 5 \quad , \quad 0.15 \leq b^x \leq 5 \end{gathered} \tag{S1}$$

for $x = \Phi$ and $x = \Theta$. Φ_0 is given below. In contrast, $0.15 \leq a^{(\Phi)} \leq 5.5$ and $0.15 \leq b^{(\Phi)} \leq 5.5$ are used in screening 1 (see details below). The range of the size parameter η is unrestricted.

Size Limits in Screenings

To avoid nanostructures of unsuitably small size, we employ constraints on the nanostructure size (i.e. size limits). This constraint is applied in the screenings, but not in the size effect studies. Nanostructures failing this constraint are assigned to zero mass activity. This constraint requires minimal length $L_{min}/2$ of the Superformula nanoshape along each positive spacial direction, so that in Eq. (4) and Eq. (8) all three constraints $\max(x) \geq L_{min}/2$ and $\max(y) \geq L_{min}/2$ and $\max(z) \geq L_{min}/2$ must be simultaneously fulfilled. Here, we use the property that the Superformulas in Eq. (4) and Eq. (8) are symmetric with respect to $x \leftrightarrow -x$, $y \leftrightarrow -y$, and $z \leftrightarrow -z$. Hence, to account for the full shape length (in positive and negative spatial direction), the size limits mentioned in the main text are given by L_{min}.

Size Constraints on Nanoparticle Centers

A second constraint requires sufficiently large nanostructure centers. This constraint is applied in the screenings, but not in the size effect studies. Nanostructures failing this constraint are assigned to zero mass activity. Two distinct centers of different sizes are considered: Within the x-y, y-z or x-z plane, a minimal number of 13 (25) atoms is required within a radius of $r_{min1} = 0.58\ nm$ ($r_{min2} = 0.82\ nm$) around the origin. For spherical-based shapes from Eq. (4), this constraint must be fulfilled on any of the x-y, y-z, and z-x plane. For cylindrical-based electrocatalysts from Eq. (8), this constraint is applied on the x-y plane only. These two different size constraints are illustrated in Figure S2.

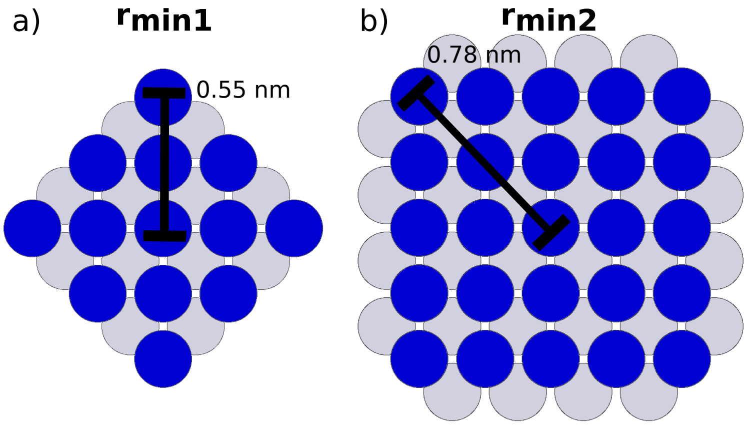

Figure S2: Size constraints on the nanostructure center are illustrated for r_{min1} (a) and r_{min2} (b). Atoms which are relevant for the size constraint are highlighted in blue.

Spherical-Based Superformula Shapes

Screening 1: S I - S V

Nanoparticles from C3 symmetric sphere-like Superformula shapes, see Eq. (4) and (6), are investigated. The parameter space in Eq. S1 is used. Minimal length $L_{min} = 3.6\ nm$ and an electrocatalyst center of type r_{min1} are required. The domain of the initial azimuthal angle is $0 \leq \Phi_0 \leq 2\pi/3$.

Note that in case the identity function $\Phi_r(\Phi) = \Phi$ and $\Phi_0 = 0$ is used, the Superformula

in Eq. (4) is periodic in the domain $\Phi \epsilon [0, \pi]$. Furthermore, the Superformula features point symmetry with respect to $\Phi = \pi/2$. Hence, the restriction of the initial azimuthal angle to $0 \leq \Phi_0 \leq 2\pi/3$, in comparison to the full domain $0 \leq \Phi_0 \leq \pi$, neglects one single nanostructure characteristic which arises at the transition between $\Phi < \pi$ and $\Phi > \pi$.

In this screening $k_{max} = 604$ iterative steps are performed. In Table S1 the Superformula parameters of electrocatalysts S V, S IV, S III, S II, and S I are listed from top row to bottom row. Those electrocatalysts are ranked the 1st, 8th, 25th, 31st, 32th most mass-active electrocatalysts in this screening.

Table S1: Superformula parameters for S V, S IV, S III, S II, and S I are listed from top row to bottom row.

η	$m^{(\Phi)}$	$n_1^{(\Phi)}$	$n_2^{(\Phi)}$	$n_3^{(\Phi)}$	$a^{(\Phi)}$	$b^{(\Phi)}$
15.0130323	8.66767693	8.56466147	1.02227019	1.01703019	0.85235843	0.95062516
$m^{(\Theta)}$	$n_1^{(\Theta)}$	$n_2^{(\Theta)}$	$n_3^{(\Theta)}$	$a^{(\Theta)}$	$b^{(\Theta)}$	Φ_0
0.72694989	7.60702259	10.3008969	9.00995688	1.3638465	3.91107538	0.3953994
η	$m^{(\Phi)}$	$n_1^{(\Phi)}$	$n_2^{(\Phi)}$	$n_3^{(\Phi)}$	$a^{(\Phi)}$	$b^{(\Phi)}$
28.8627798	0.15	2.51710177	4.10462116	10.134377	0.8499274	2.19746894
$m^{(\Theta)}$	$n_1^{(\Theta)}$	$n_2^{(\Theta)}$	$n_3^{(\Theta)}$	$a^{(\Theta)}$	$b^{(\Theta)}$	Φ_0
0.65994789	5.43739073	11.5592702	2.67341226	0.98460203	2.40358451	0.52440039
η	$m^{(\Phi)}$	$n_1^{(\Phi)}$	$n_2^{(\Phi)}$	$n_3^{(\Phi)}$	$a^{(\Phi)}$	$b^{(\Phi)}$
0.33056636	4.53379749	7.34676495	10.0152075	9.36374397	0.90203455	1.54749415
$m^{(\Theta)}$	$n_1^{(\Theta)}$	$n_2^{(\Theta)}$	$n_3^{(\Theta)}$	$a^{(\Theta)}$	$b^{(\Theta)}$	Φ_0
1.16255985	1.57799768	11.4170086	5.20363299	1.67461609	4.60247524	1.43023487
η	$m^{(\Phi)}$	$n_1^{(\Phi)}$	$n_2^{(\Phi)}$	$n_3^{(\Phi)}$	$a^{(\Phi)}$	$b^{(\Phi)}$
16.7387325	2.61972749	2.46718322	0.39838442	6.42340979	3.04636248	0.63708718
$m^{(\Theta)}$	$n_1^{(\Theta)}$	$n_2^{(\Theta)}$	$n_3^{(\Theta)}$	$a^{(\Theta)}$	$b^{(\Theta)}$	Φ_0
0.36310486	7.45835475	10.3219763	8.96007665	1.36280679	3.82813315	0.62052876
η	$m^{(\Phi)}$	$n_1^{(\Phi)}$	$n_2^{(\Phi)}$	$n_3^{(\Phi)}$	$a^{(\Phi)}$	$b^{(\Phi)}$
17.2883478	9.85879152	8.55624214	2.95718801	0.15	1.36499404	1.13672796
$m^{(\Theta)}$	$n_1^{(\Theta)}$	$n_2^{(\Theta)}$	$n_3^{(\Theta)}$	$a^{(\Theta)}$	$b^{(\Theta)}$	Φ_0
2.64976382	6.65443915	6.81113644	3.75219949	1.18095185	1.52034653	1.69209911

Screening 2: S VI

Nanoparticles from C3 symmetric sphere-like shapes, see Eq. (4) and (6), are investigated. The parameter space in Eq. S1 is used, except of $0.15 \leq a^{(\Phi)} \leq 5.5$ and $0.15 \leq b^{(\Phi)} \leq 5.5$. The domain of the initial azimuthal angle is $0 \leq \Phi_0 \leq \pi$. Minimal length $L_{min} = 3\ nm$ and an electrocatalyst center of type r_{min1} are required. $k_{max} = 800$ iterative steps are performed. Electrocatalyst S VI has the highest mass activity in this screening. Associated Superformula parameters are given in Table S2.

Table S2: Superformula parameters for S VI.

η	$m^{(\Phi)}$	$n_1^{(\Phi)}$	$n_2^{(\Phi)}$	$n_3^{(\Phi)}$	$a^{(\Phi)}$	$b^{(\Phi)}$
9.24986209	3.42626465	5.99921655	1.22137537	0.26164179	4.36075805	4.81546432
$m^{(\Theta)}$	$n_1^{(\Theta)}$	$n_2^{(\Theta)}$	$n_3^{(\Theta)}$	$a^{(\Theta)}$	$b^{(\Theta)}$	Φ_0
2.13570347	7.90612175	10.6103113	9.49802803	1.51595124	4.7888501	1.87136639

Cylindrical-Based Superformula Shapes

Screening 3: C I, C II, C IV - C VII

Nanostructures from C4 symmetric rod-like shapes, see Eq. (7) and (8), are investigated. Minimal length $L_{min} = 1.8\ nm$, and an electrocatalyst center of type r_{min2} are required. The domain of the initial azimuthal angle is $0 \leq \Phi_0 \leq 3\pi/4$ which is a restriction of the full domain $0 \leq \Phi_0 \leq \pi$ as discussed for screening 1 above. In this screening $k_{max} = 725$ iterative steps are performed. In Table S3 the Superformula parameters of electrocatalysts C VII, C VI, C V, C IV, C II, and C I are listed from top row to bottom row. Those electrocatalysts are ranked the 8th, 10th, 16th, 18th, 35th, and 39th most mass-active electrocatalysts in this screening.

Table S3: Superformula parameters for C VII, C VI, C V, C IV, C II, and C I are listed from top to bottom.

η	$m^{(\Phi)}$	$n_1^{(\Phi)}$	$n_2^{(\Phi)}$
1.11680704	8.13490331	1.66128915	4.69137758
$n_3^{(\Phi)}$	$a^{(\Phi)}$	$b^{(\Phi)}$	Φ_0
7.18440609	2.43945087	1.70022311	8.27169793e-02

η	$m^{(\Phi)}$	$n_1^{(\Phi)}$	$n_2^{(\Phi)}$
9.51421522e-01	8.29924038	1.48262128	4.81676836
$n_3^{(\Phi)}$	$a^{(\Phi)}$	$b^{(\Phi)}$	Φ_0
7.11093190	2.28579417	1.69242114	4.27290983e-02

η	$m^{(\Phi)}$	$n_1^{(\Phi)}$	$n_2^{(\Phi)}$
1.26001236	8.09301960	1.54759006	4.64105465
$n_3^{(\Phi)}$	$a^{(\Phi)}$	$b^{(\Phi)}$	Φ_0
7.29454620	2.20140982	1.67816996	1.97127645e-02

η	$m^{(\Phi)}$	$n_1^{(\Phi)}$	$n_2^{(\Phi)}$
5.42562388e-01	8.02454194	1.28964584	4.34517632
$n_3^{(\Phi)}$	$a^{(\Phi)}$	$b^{(\Phi)}$	Φ_0
7.61563429	2.26257106	1.78334607	2.30115820

η	$m^{(\Phi)}$	$n_1^{(\Phi)}$	$n_2^{(\Phi)}$
5.34890168	6.03415982	2.19913750	3.42445165
$n_3^{(\Phi)}$	$a^{(\Phi)}$	$b^{(\Phi)}$	Φ_0
8.52525678	2.09285952	1.09325492	1.32528849

η	$m^{(\Phi)}$	$n_1^{(\Phi)}$	$n_2^{(\Phi)}$
8.73262651	8.89684815	9.61971640	3.03333679
$n_3^{(\Phi)}$	$a^{(\Phi)}$	$b^{(\Phi)}$	Φ_0
8.57440225	1.83069690	4.74192482	1.57973673

Screening 4: C XI

Nanostructures from C3 symmetric rod-like shapes, see Eq. (6) and (8), are investigated. No minimal length, i.e. $L_{min} = 0\ nm$, and an electrocatalyst center of type r_{min1} are required. The domain of the initial azimuthal angle is $0 \leq \Phi_0 \leq \pi$. This screening performs $k_{max} = 589$ iterative steps. Electrocatalyst C XI has the highest mass activity in this screening. Associated Superformula parameters are given in Table S4.

Table S4: Superformula parameters of C XI.

η	$m^{(\Phi)}$	$n_1^{(\Phi)}$	$n_2^{(\Phi)}$
9.30713379	10.6220048	10.825412	5.72623315
$n_3^{(\Phi)}$	$a^{(\Phi)}$	$b^{(\Phi)}$	Φ_0
8.76428427	2.95427703	1.09599458	0.61712816

Screening 5: C III, C VIII - C XII

Nanostructures from C4 symmetric rod-like shapes, see Eq. (7) and (8), are investigated. No minimal length, i.e. $L_{min} = 0\ nm$, and an electrocatalyst center of type r_{min1} are required. The domain of the initial azimuthal angle is $0 \leq \Phi_0 \leq \pi$. In this screening $k_{max} = 800$ iterative steps are performed. In Table S2 the Superformula parameters of electrocatalysts C XII, C X, C IX, C VIII, and C III are listed from top row to bottom row. Those electrocatalysts are ranked the 1st, 22th, 28th, 29th, and 47th most mass-active electrocatalyst in this screening.

Table S5: Superformula parameters for C XII, C X, C IX, C VIII, and C III are listed from top row to bottom row.

η	$m^{(\Phi)}$	$n_1^{(\Phi)}$	$n_2^{(\Phi)}$
6.34058587	6.72415228	5.55889465	5.57073145
$n_3^{(\Phi)}$	$a^{(\Phi)}$	$b^{(\Phi)}$	Φ_0
8.84815205	0.77775366	1.69404102	0.24748348
η	$m^{(\Phi)}$	$n_1^{(\Phi)}$	$n_2^{(\Phi)}$
5.98466811	5.05778649	6.68133057	6.88066407
$n_3^{(\Phi)}$	$a^{(\Phi)}$	$b^{(\Phi)}$	Φ_0
8.86392613	0.7603646	2.03860911	3.14159265
η	$m^{(\Phi)}$	$n_1^{(\Phi)}$	$n_2^{(\Phi)}$
6.22206452	7.41058419	4.93061999	7.00810509
$n_3^{(\Phi)}$	$a^{(\Phi)}$	$b^{(\Phi)}$	Φ_0
8.7331901	0.82017299	1.68971489	0.50360082
η	$m^{(\Phi)}$	$n_1^{(\Phi)}$	$n_2^{(\Phi)}$
7.9426879	6.07555227	7.57268235	4.35372547
$n_3^{(\Phi)}$	$a^{(\Phi)}$	$b^{(\Phi)}$	Φ_0
9.80301762	1.08893828	3.02104762	0.5733896
η	$m^{(\Phi)}$	$n_1^{(\Phi)}$	$n_2^{(\Phi)}$
0.21170728	8.99991203	0.57896453	11.88102878
$n_3^{(\Phi)}$	$a^{(\Phi)}$	$b^{(\Phi)}$	Φ_0
6.725375	0.5091187	1.47841636	2.94862459

Nanostructure Size Effects

In the main text, nanostructure size effects on the catalytic mass activities are examined for S II, S V, S VI, C VII, C XI, and C XII. All nanostructures, which emerge in these studies, are presented below in Figures S3, S5, S7, S9, S11, and S13. Note that ascending label numbers correspond to an increasing size parameter η in the Superformula.

Furthermore, the $\overline{CN}$ distribution for a selection of those nanostructures is provided in Figures S4, S6, S8, S10, S12, and S14. Note that atoms with $\overline{CN} > 10$ are not included in the figures.

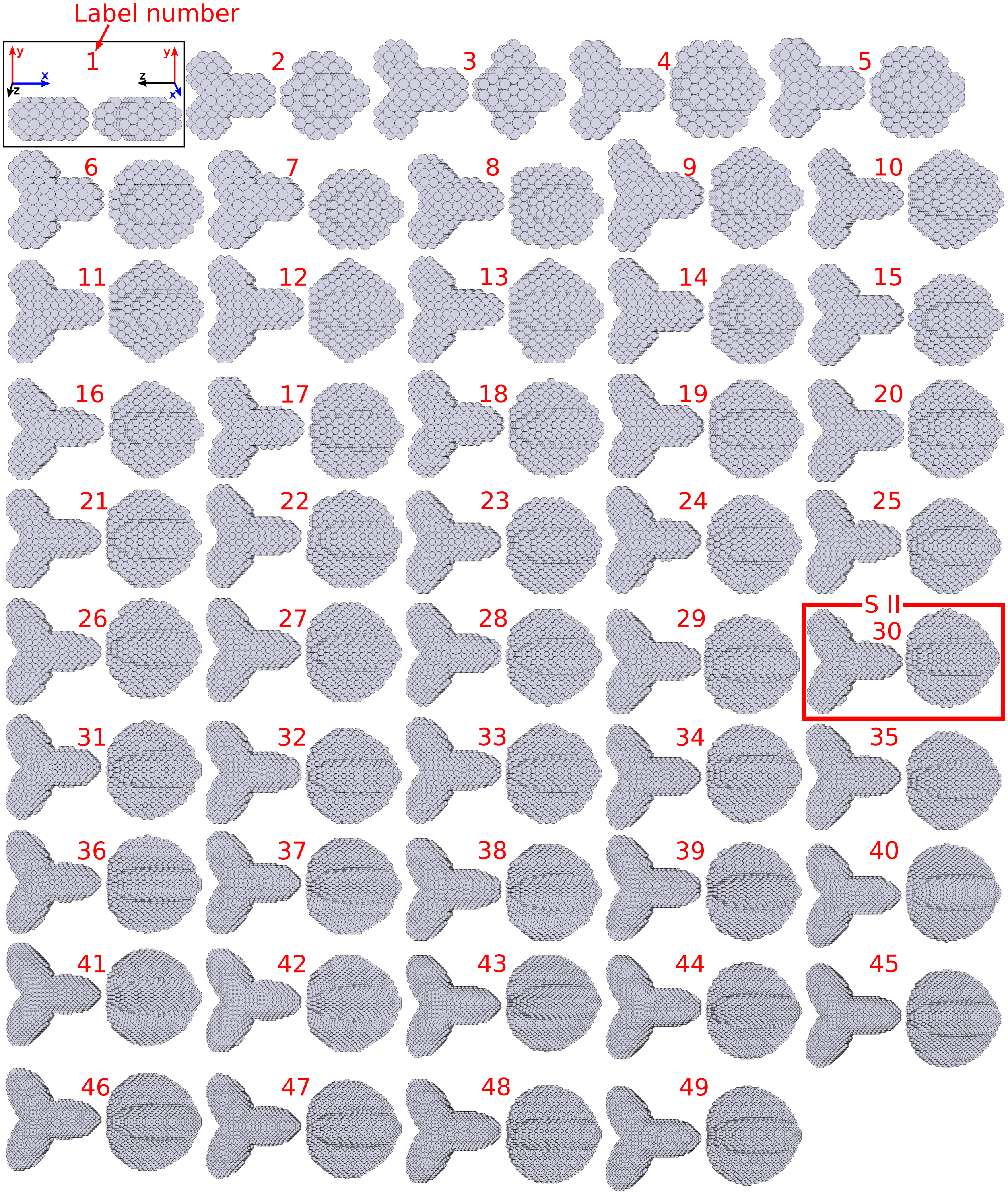

Figure S3: Electrocatalysts are shown which are involved in the nanostructure size effect study for S II.

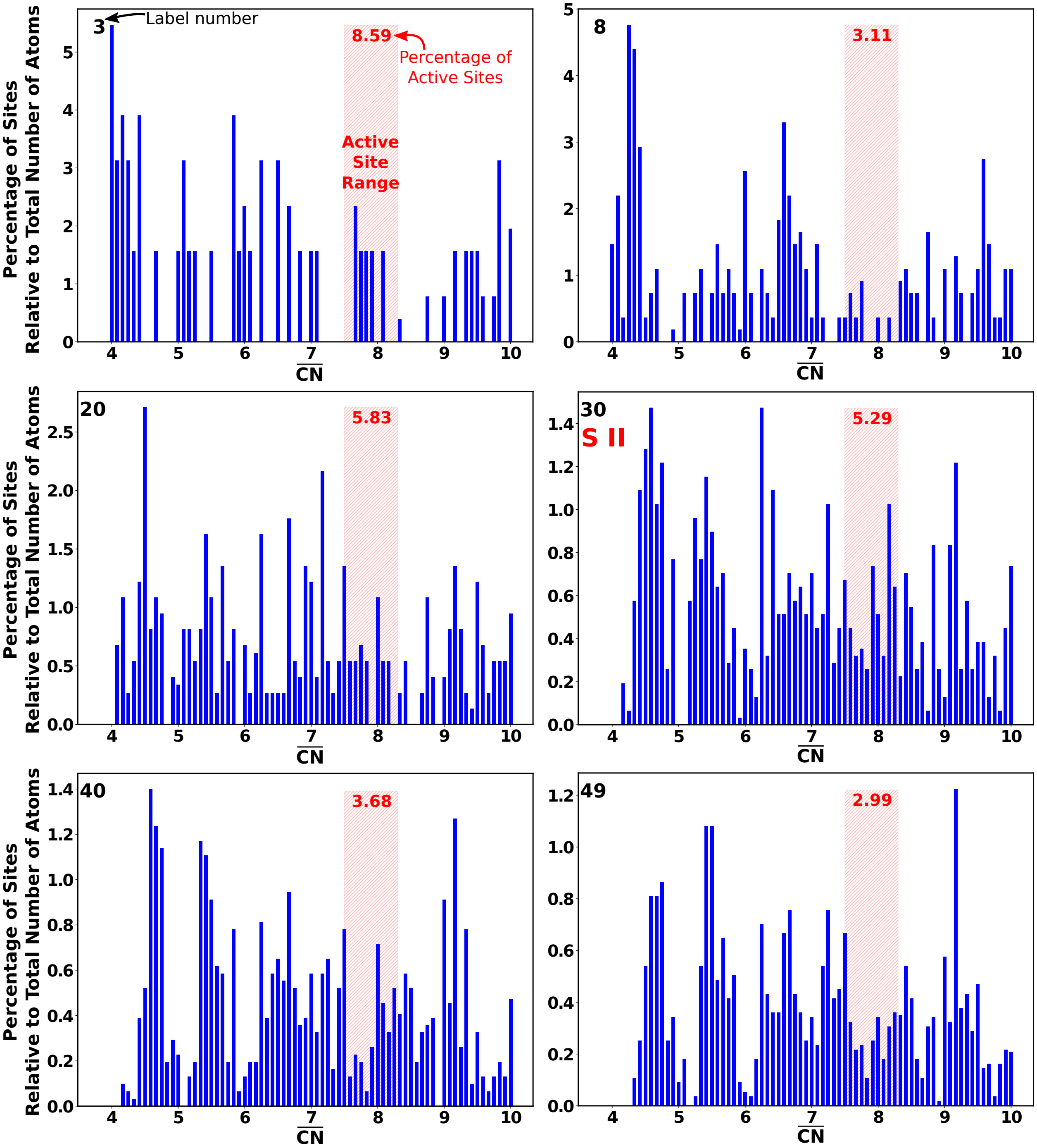

Figure S4: $\overline{CN}$ distribution of electrocatalysts 3, 8, 20, 30, 40, and 49 shown in Figure S3. The red highlighted area illustrates the active site range $7.5 \leq \overline{CN} \leq 8.3$. The percentage of active sites with $7.5 \leq \overline{CN} \leq 8.3$ is printed in red. Note that atoms with $\overline{CN} > 10$ are not included in the plots.

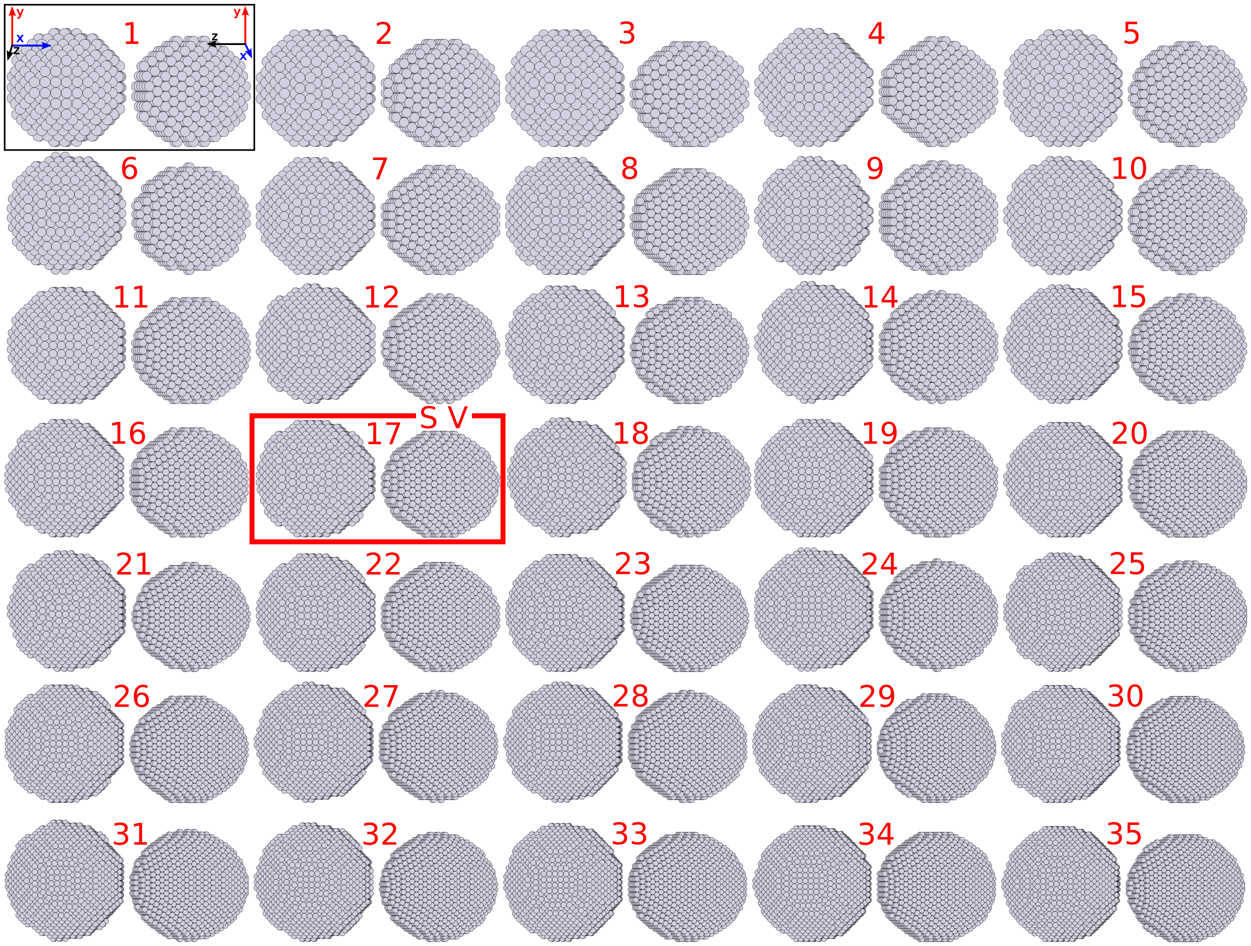

Figure S5: Electrocatalysts are shown which are involved in the nanostructure size effect study for S V.

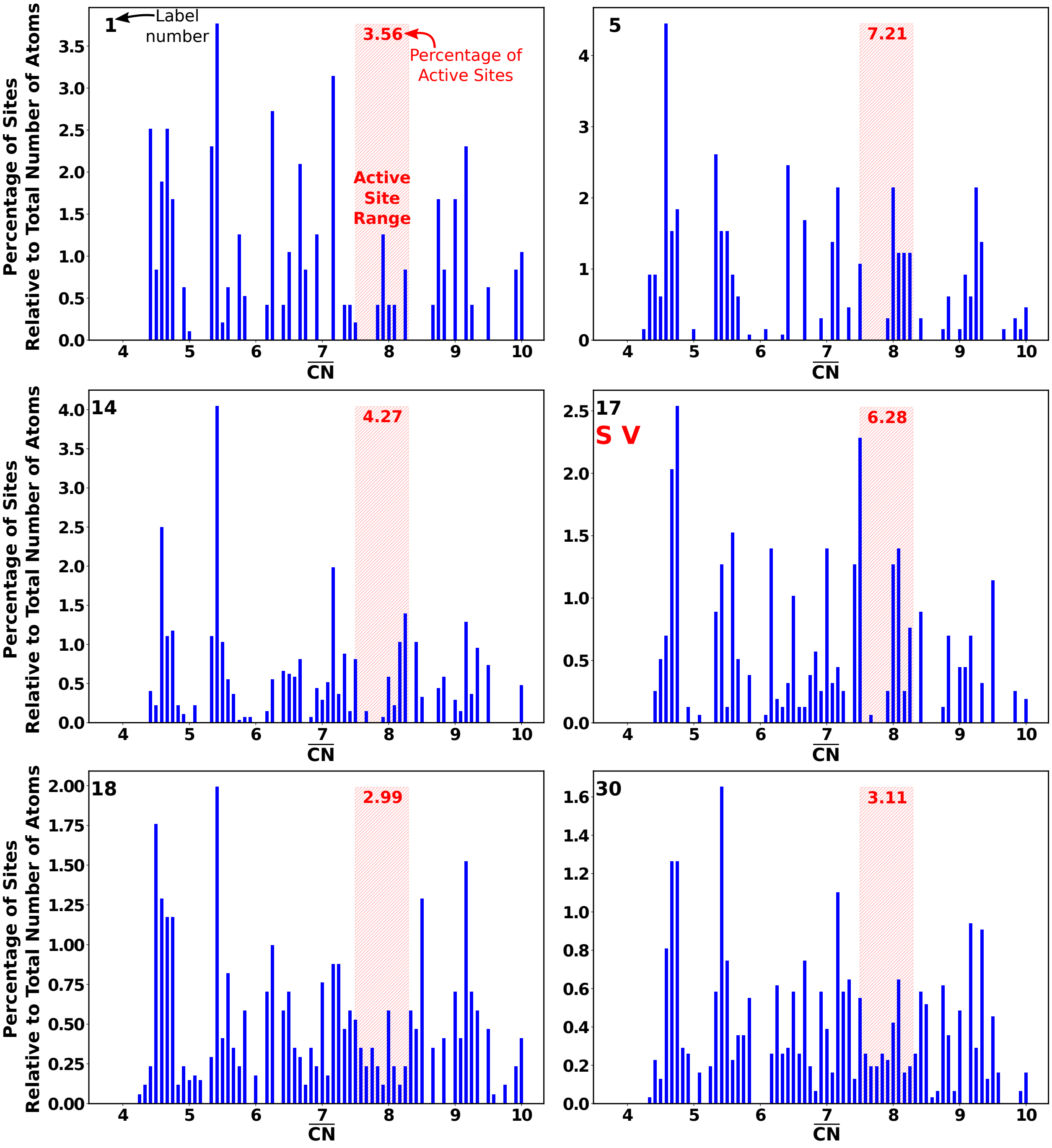

Figure S6: $\overline{CN}$ distribution of electrocatalysts 1, 5, 14, 17, 18, and 30 shown in Figure S5. Note that atoms with $\overline{CN} > 10$ are not included in the plots.

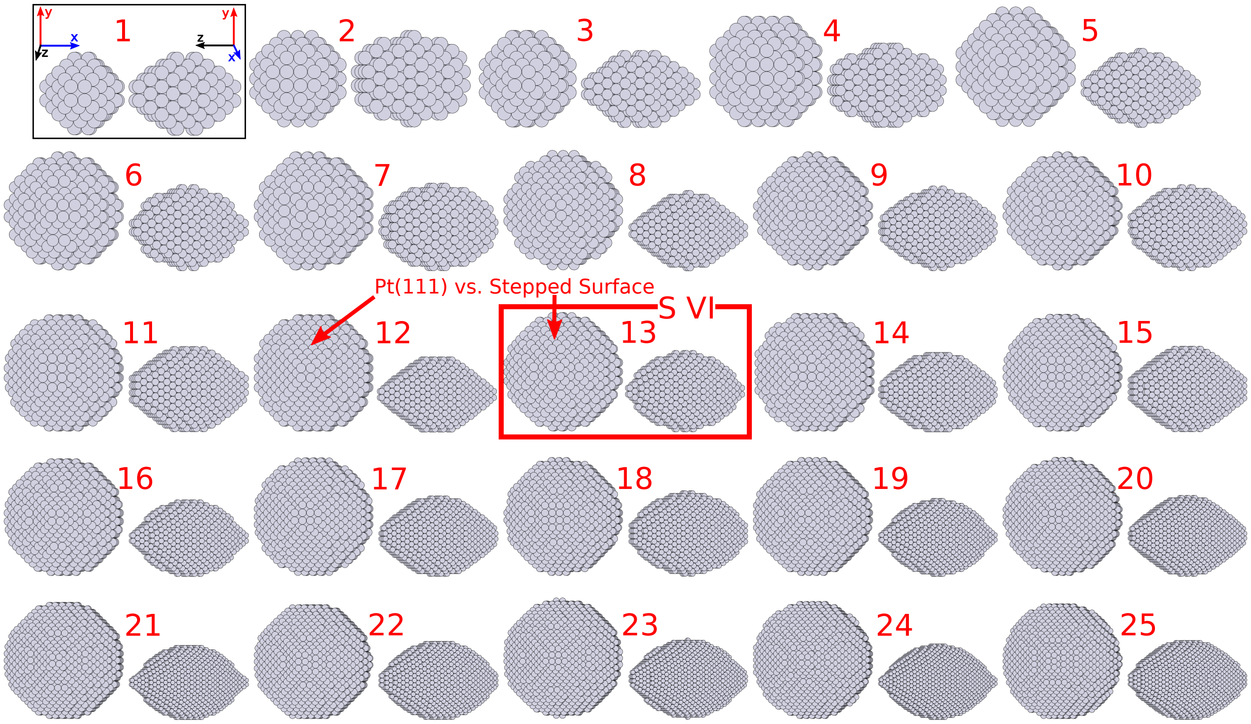

Figure S7: Electrocatalysts are shown which are involved in the nanostructure size effect study for S VI. Note that the optimized shape S VI has a high $\overline{CN}$ distribution near the optimal value $\overline{CN} = 8.1$ (see Figure S8), while less active Pt(111) sites dominate on electrocatalyst 12 of similar size.

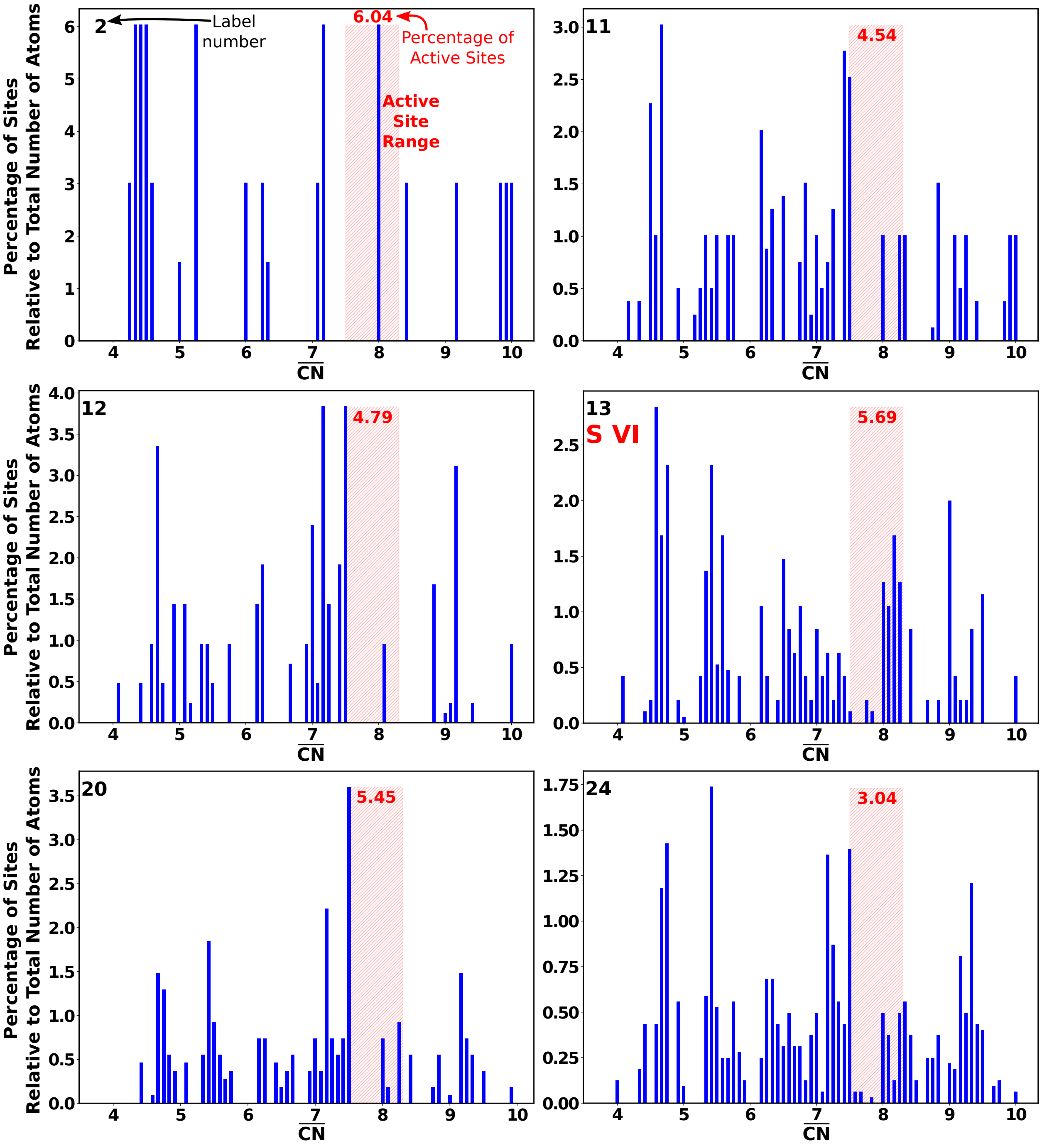

Figure S8: $\overline{CN}$ distribution of electrocatalysts 2, 11, 12, 13, 20, and 24 shown in Figure S7. Note that atoms with $\overline{CN} > 10$ are not included in the plots.

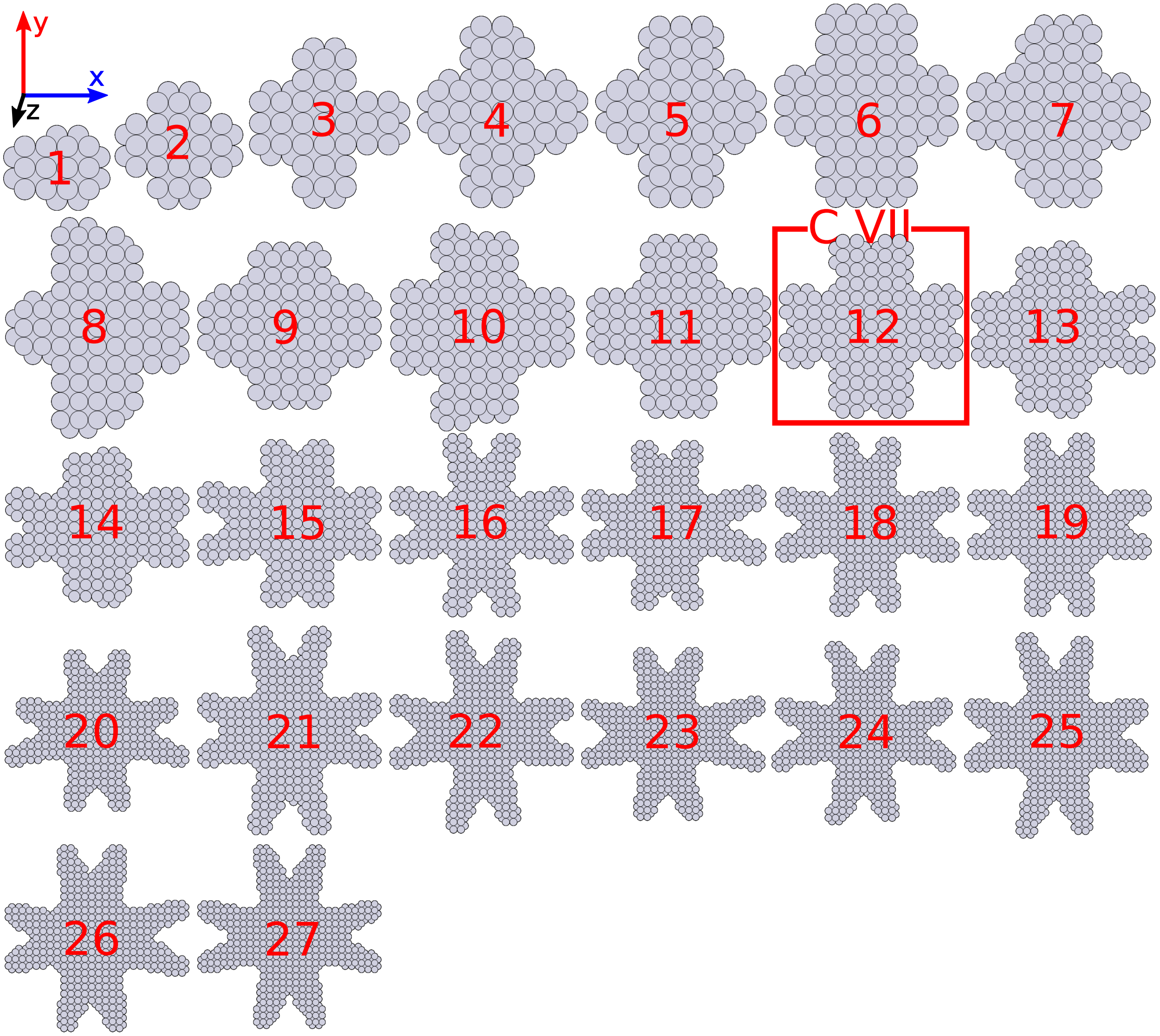

Figure S9: Electrocatalysts are shown which are involved in the nanostructure size effect study for C VII.

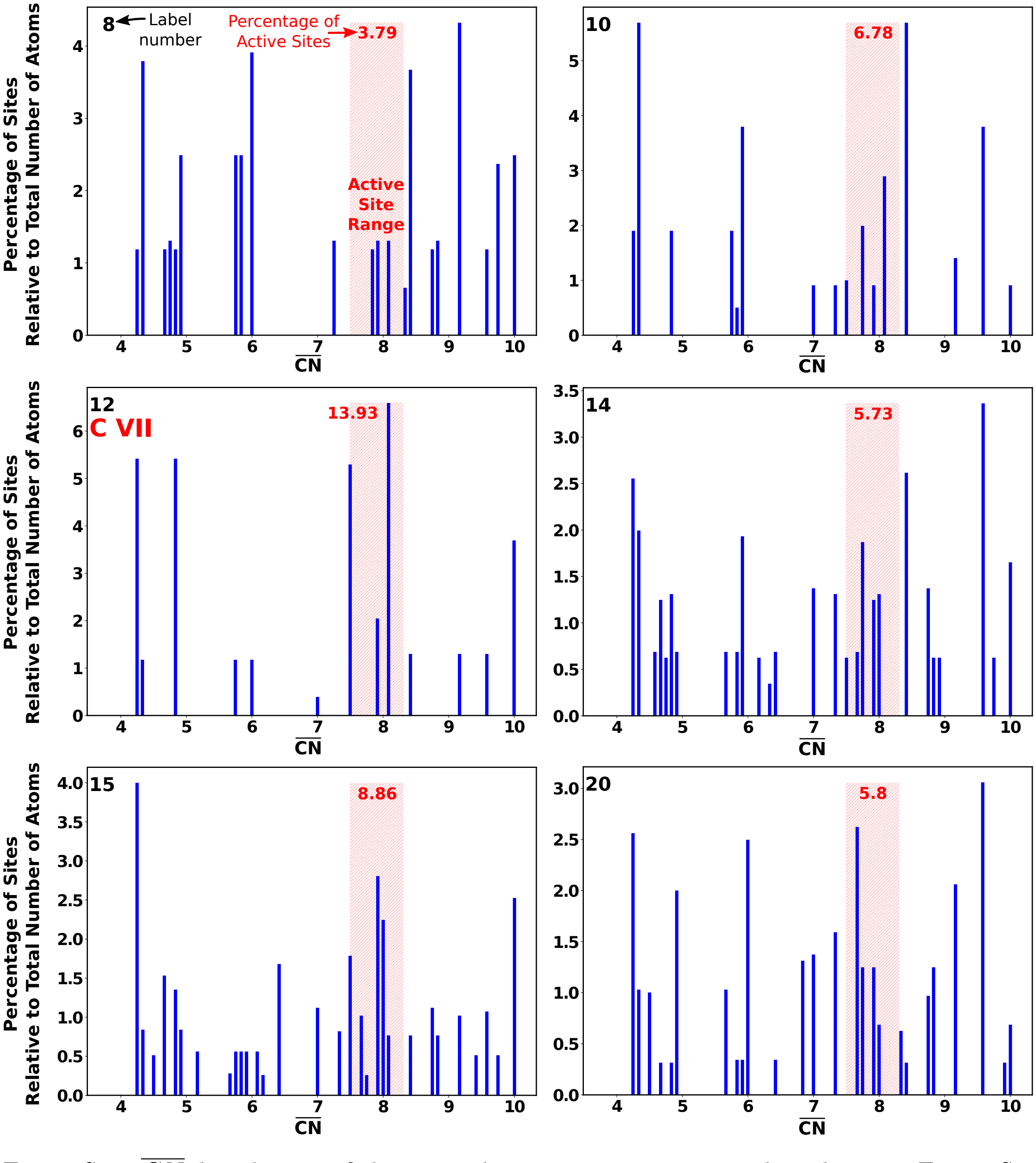

Figure S10: $\overline{CN}$ distribution of electrocatalysts 8, 10, 12, 14, 15, and 20 shown in Figure S9. Note that atoms with $\overline{CN} > 10$ are not included in the plots.

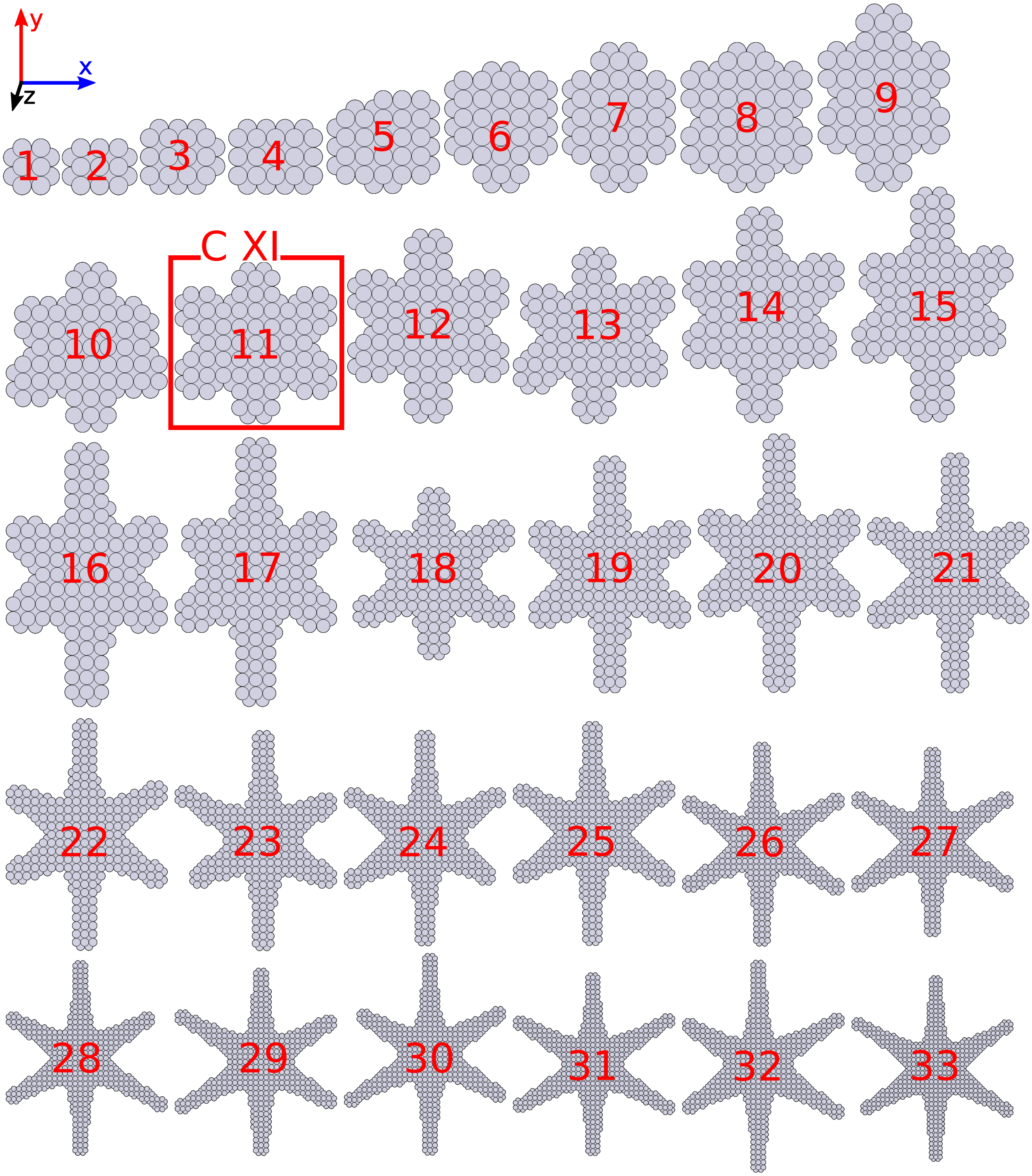

Figure S11: Electrocatalysts are shown which are involved in the nanostructure size effect study for C XI.

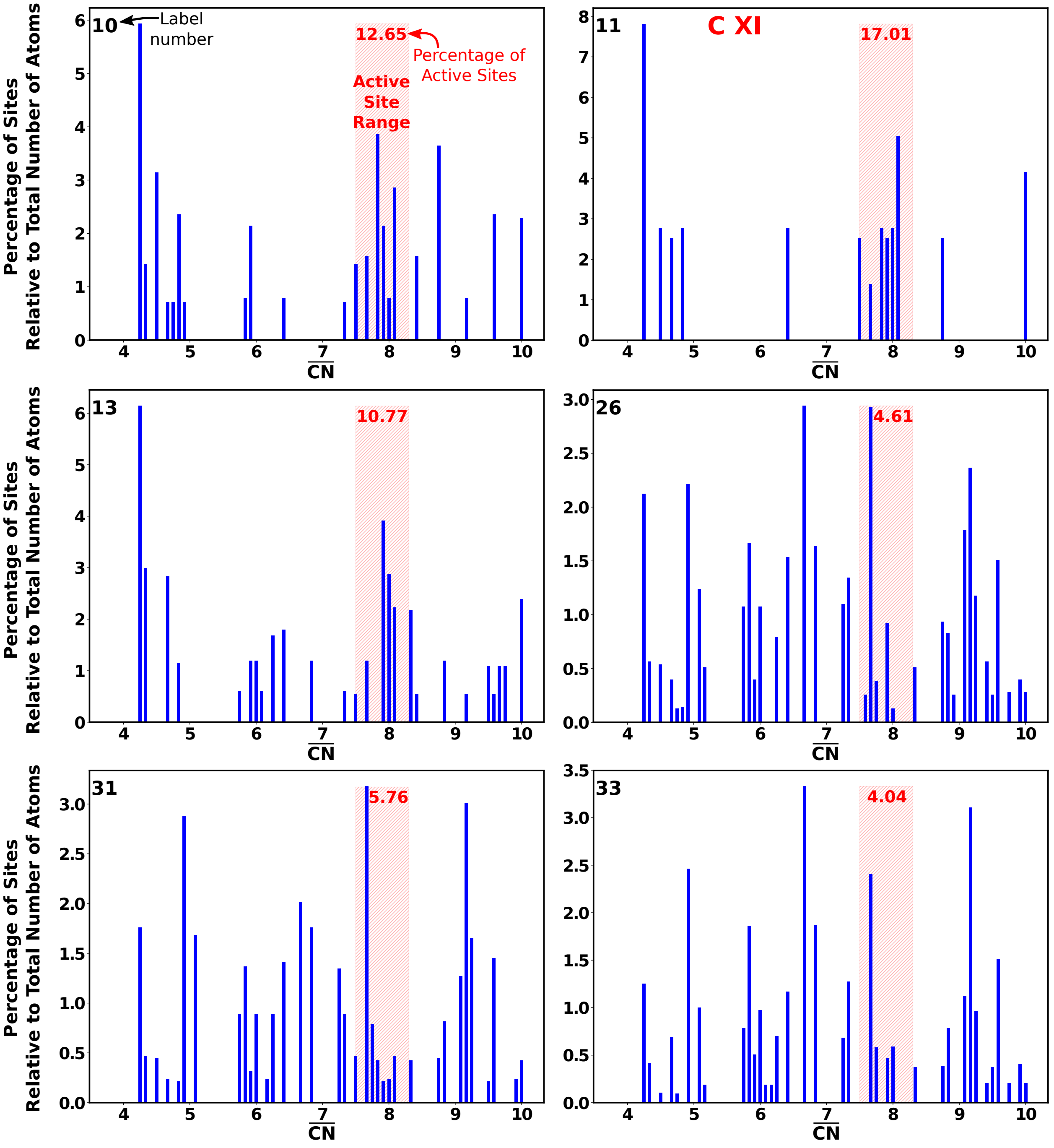

Figure S12: $\overline{CN}$ distribution of electrocatalysts 10, 11, 13, 26, 30, 31, and 33 shown in Figure S11. Note that atoms with $\overline{CN} > 10$ are not included in the plots.

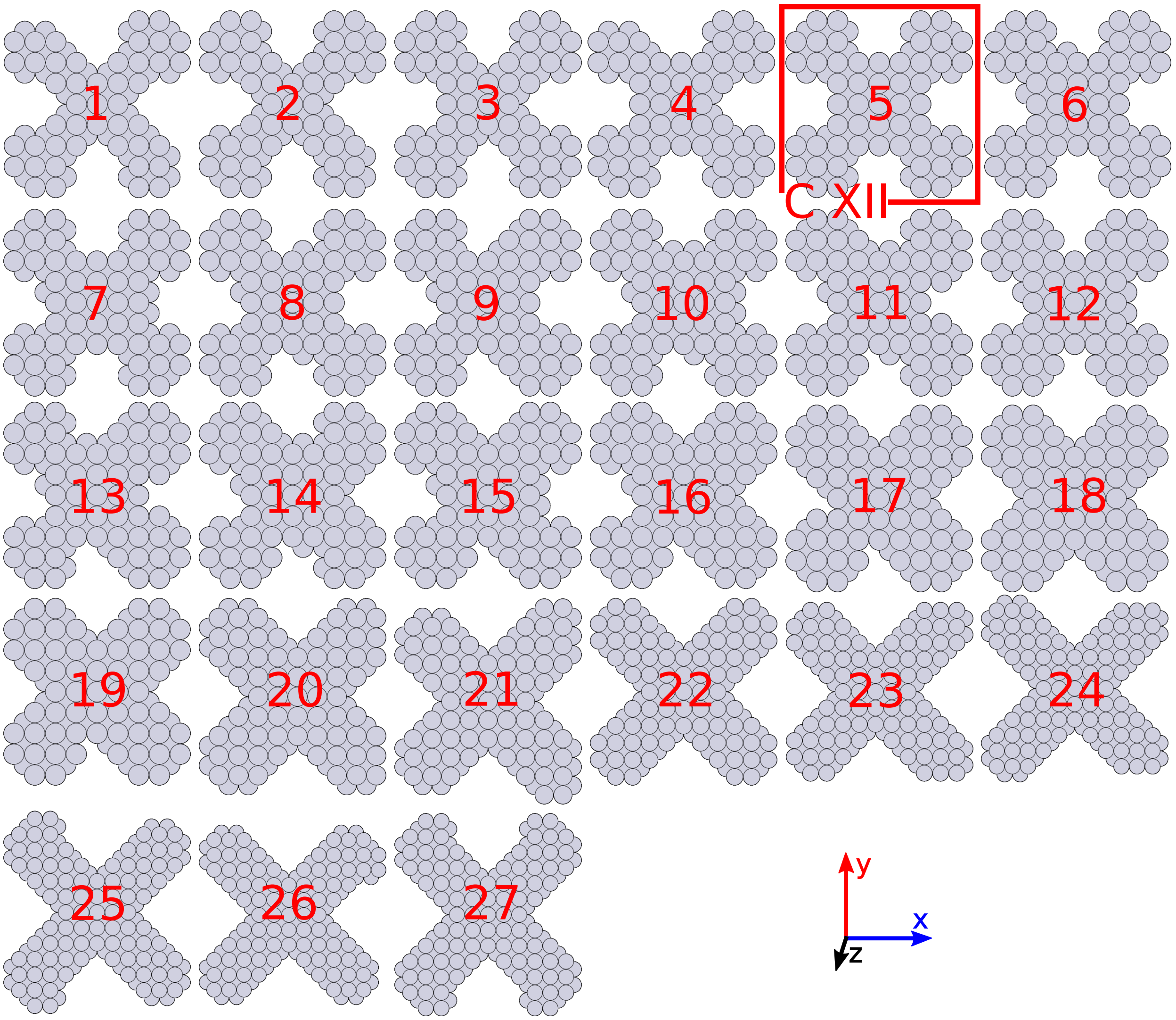

Figure S13: Electrocatalysts are shown which are involved in the nanostructure size effect study for C XII.

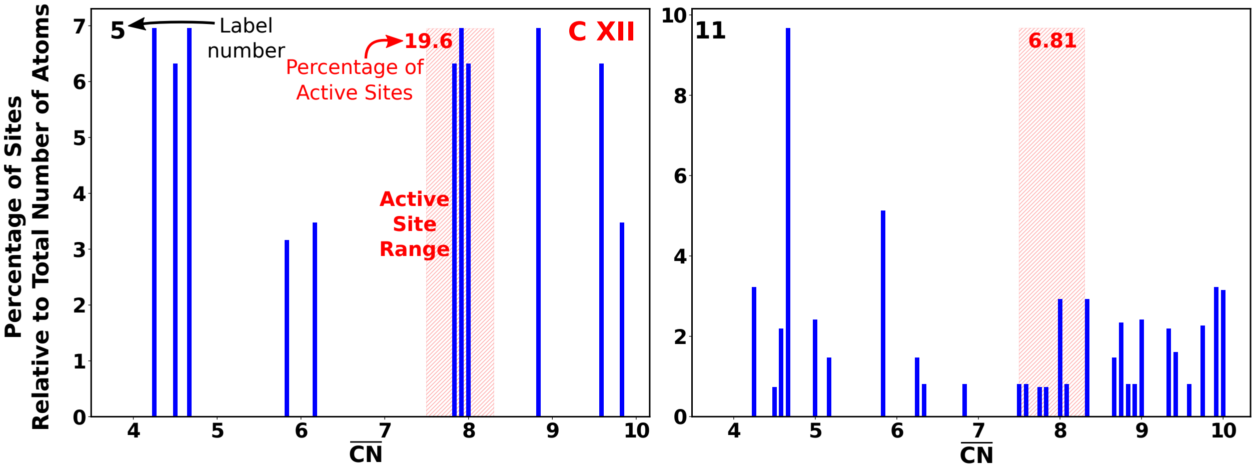

Figure S14: $\overline{CN}$ distribution of electrocatalysts 5 and 11 shown in Figure S13. Note that atoms with $\overline{CN} > 10$ are not included in the plots.
